# Clinical Chemistry and Hematology Values in Captive European Mink (*Mustela lutreola*): Reference Intervals and Evaluation of the Effects of Sex, Age, and Body Condition

**DOI:** 10.3390/vetsci13070619

**Published:** 2026-06-26

**Authors:** Mª Carmen Aranda, Paloma Jimena de Andrés, Sergio Villanueva-Saz, Mª de los Ángeles Jiménez

**Affiliations:** 1 Department of Medicine and Surgery, Veterinary Faculty, Complutense University of Madrid, 28040 Madrid, Spain or maranda4@tragsa.es (M.C.A.); mariadji@ucm.es (M.d.l.Á.J.); 2 Tragsatec, 28037 Madrid, Spain; 3 FIEB Foundation (Foundation for Research in Ethology and Biodiversity), 45950 Toledo, Spain; 4 Animal Pathology Department and Clinical Immunology Laboratory, Veterinary Faculty, Zaragoza University, 50005 Zaragoza, Spain; svs@unizar.es

**Keywords:** European mink, hematology, serum biochemistry, reference intervals, conservation medicine, health monitoring, mustelids

## Abstract

The European mink is a critically endangered mustelid. Species-specific hematological and biochemical reference intervals are lacking, complicating accurate health assessments in conservation programs. This study aimed to determine these reference intervals for European mink, following international guidelines, with 110 blood samples collected under isoflurane anesthesia, outside the breeding season, from 75 captive individuals (37 females, 38 males). Results were broadly comparable to the American mink and domestic ferret. Several parameters varied significantly according to sex, age, and body condition. Males showed higher hemoglobin (*p* = 0.031), whereas females, higher platelet (*p* = 0.014) and leukocyte counts, cholesterol, urea, alkaline phosphatase (ALP) (*p* ≤ 0.002), and globulin concentrations (*p* = 0.005). Males also had higher creatinine and alanine aminotransferase (ALT) (*p* < 0.001). Juveniles had higher leukocyte counts (*p* = 0.047) and ALP and calcium concentrations (*p* ≤ 0.016), while adults showed higher globulin and creatinine values (*p* ≤ 0.014). Overweight individuals exhibited increased hemoglobin and hematocrit (*p* ≤ 0.018) and mild electrolyte differences (*p* ≤ 0.045). Separate intervals for sex and age were not warranted for most analytes. These results provided specific reference intervals useful for essential health monitoring and clinical decision-making in conservation breeding, translocation, and reintroduction programs of European mink.

## Simple Summary

The European mink is one of the most endangered mammals in Europe, and effective conservation strategies require reliable tools for health assessment. The purpose of this study is to establish hematological and serum biochemical reference intervals for healthy captive European mink and determine the effects of sex, age, and body condition. Overall, blood profiles in the European mink were comparable to those of other mustelids such as American mink and ferrets, and sex- and age-related differences were identified. Variations associated with growth and immune development were recorded in juveniles, while body condition modestly influenced selected variables. These results provide baseline data essential to support health monitoring and management of conservation breeding, translocation, and reintroduction programs.

## 1. Introduction

The European mink (*Mustela lutreola*) is a small semi-aquatic carnivore and one of the most endangered mammals in Europe, according to the International Union for Conservation of Nature [1]. It is currently listed under Annexes II and IV of the Habitats Directive and Appendix II of the Bern Convention as a strictly protected animal species [2]. The dramatic decline of the European mink population is mainly driven by habitat loss and degradation, human-related mortality, competition with the invasive American mink (*Neogale vison*) and infectious diseases, among other causes [1,3,4,5,6]. Several pathogens have been identified in the European mink, considered potential threats for survival [7,8].

The estimated population of European mink in 2003 in Spain was less than 500 individuals [1,9]; however, recent estimations indicate that less than 157 individuals remain in the wild [10,11]. In response to this decline, Spanish authorities have implemented significant conservation strategies aimed to safeguard the species in the wild, that include measures to reduce non-natural mortality, ensure genetic preservation, prevent and control diseases and epizootics, and eliminate feral populations of American mink, and have developed a national ex situ conservation program [3,9]. The Foundation for Research in Ethology and Biodiversity (FIEB) is one of three European mink captive breeding centers authorized in Spain by the Ministry for the Ecological Transition and the Demographic Challenge within the national conservation ex situ program [3,11] and holds the largest population of European mink in captivity in Europe. The FIEB center focuses on the breeding and maintenance of genetically valuable individuals in a controlled environment that enables natural behaviors and animal welfare. In addition to reproduction, the FIEB center provides animals for reintroduction and population reinforcement programs coordinated at the national level. The facility also supports research and monitoring activities related to health, behavior, and husbandry, generating scientific information essential for the improvement of management protocols and conservation outcomes [11]. Currently, the national captive breeding program harbors 65 individuals in captivity (unpublished data). These actions are part of a coordinated national strategy designed to ensure the long-term viability of the species [3,9,11].

Like many other wild mammals, European mink rarely show clinical signs until late stages of disease [12]. Consequently, the early detection and management of health issues are essential to ensure optimal care and welfare in captive wild animals [13]. Routine health examinations, including physical assessments and blood analyses, enable early detection of disease or potential health disorders in captive populations [14,15,16]. Similarly, the incorporation of blood sampling during routine captures in the wild would be an effective tool for health monitoring of the free-ranging population. However, this approach is currently limited due to the lack of species-specific reference intervals, and health assessments often rely on extrapolation of data from related species [14].

Hematology and blood chemistry data from clinically healthy individuals are therefore essential in order to establish reliable baseline parameters for the species. The panel should comprise analytes that serve as indicators of major disruptions in homeostasis, metabolic alterations, and organ damage or dysfunction [14]. Key analytes are complete blood counts (CBCs), electrolytes, metabolic indicators (glucose, cholesterol, triglycerides), and organ function markers. Commonly evaluated enzymes include alanine aminotransferase (ALT), aspartate aminotransferase (AST), alkaline phosphatase (ALP), gamma-glutamyl transferase (GGT), glutamate dehydrogenase (GLDH) and creatine kinase (CK), which assess hepatic and muscular integrity. Additional biomarkers such as amylase and vitamin E provide information on metabolic health and reproductive function, as vitamin E influences antioxidant balance and sperm quality [17,18,19].

Hematological and biochemical reference intervals have been reported for other mustelids, such as the American mink [20,21] and domestic ferret [22,23,24], but species-specific physiological differences limit their direct applicability to the European mink. The acquisition of clinical laboratory data from healthy European mink maintained in a controlled environment is a valuable resource for establishing reliable baseline hematological and biochemical parameters. These reference values can then be applied as a basis for accurate health assessments in free-ranging individuals. Reference values for serum protein fractions in European mink have been previously established by our group, using cellulose acetate and agarose gel electrophoresis [25]. These previously reported findings are complementary to the present study, which expands the dataset of available hematological and biochemical analytes.

The aim of this study was to establish baseline hematological and biochemical reference intervals for healthy European mink using a captive population maintained in a controlled, healthy environment, and assess the existence of physiological variations amongst these analytes based on sex, age, and body condition. This data will provide a solid reference for health and disease monitoring of the wild population and support conservation efforts.

## 2. Materials and Methods

### 2.1. Animals and Study Site

The study was carried out amongst the captive population of European mink (*Mustela lutreola*) safeguarded by the Spanish Ex Situ Conservation Program at the FIEB center in Casarrubios del Monte (Toledo, Spain). All animals housed at the facility were part of this conservation program. Sample individuals were captive-born which allowed accurate traceability of identification and age. All animals were identified with a microchip one month after birth. Only sexually mature animals, 8 months and older, were included in the study, and all individuals were sampled during the non-breeding season (August–February) as part of the program’s monitoring routine [26]. In accordance with established management protocols, no pregnant or lactating females were sampled to avoid any interference with breeding and rearing. Two age groups were established: juveniles (8–12 months) and adults (>12 months) [27].

Animals were submitted to routine, periodic health checks and monitored for wellbeing. Daily visual inspections, fortnight weight checks, routine deworming protocols for endo- and ectoparasites were carried out by specialized personnel.

Mink were housed individually in outdoor enclosures, delimited by wire mesh fencing, which enabled exposure to natural environmental conditions such as ventilation, temperature and photoperiod. Three types of enclosures were available according to size and dimensions: 35 m^2^ with a 5 × 1 × 0.7 m pool (*n* = 10), 55 m^2^ with a 5 × 3 × 0.7 m pool (*n* = 16), and 95 m^2^ with a 10 × 3 × 0.7 m pool (*n* = 2). All pools had continuous running water, allowing animals to perform natural swimming behavior.

Each enclosure contained trees, shrubs and aquatic vegetation, providing a heterogeneous environment with vegetated areas interspersed with open spaces. Structural enrichment included at least one nest box (27 × 79 × 30 cm) as well as additional shelters constructed from plastic tunnels, logs and branches. A roofed area provided shade. In addition, enclosures were equipped with an overhead irrigation system that generated artificial rainfall during hot periods to maintain humidity and improve thermal comfort.

Individuals were periodically rotated among enclosures during their stay at the facility to increase environmental variability and promote enrichment. Additional transfers between enclosures occurred during the breeding season as part of reproductive management.

Animals received a weekly rotating diet with day to day variations designed to resemble the natural prey spectrum and stimulate predatory behavior. Each individual was fed one live Japanese quail (*Coturnix japonica*), one dead quail, two one-day-old chicks, two trout, one live mouse/rat per week. Live mice and rats were bred on-site at the FIEB facilities, frozen food items were obtained from a commercial supplier specialized in the production and distribution of frozen feed for animal consumption, operating under applicable European animal health and feed safety regulations and providing full batch traceability. Although frozen storage may be associated with gradual degradation of certain labile nutrients, including vitamin E and thiamine, all products were stored and handled according to the manufacturer’s recommendations and used within their specified shelf life. Live Japanese quail were obtained from a licensed commercial breeder operating under national animal welfare and biosecurity regulations. Birds were sourced from a monitored production facility subject to routine veterinary oversight and sanitary controls, including screening for relevant avian pathogens such as *Salmonella* spp.

In addition to the standard feeding regime, animals participated in a daily environmental enrichment program, which included the provision of live prey such as crucian carp (*Carassius* sp.), red swamp crayfish (*Procambarus clarkii*) or eggs, as well as feeding devices used to hide food, and structural enrichment such as hammocks, promoting exploratory, resting and play behaviors.

All animal care and husbandry procedures complied with national regulations and institutional animal welfare protocols [3,28] and were conducted by trained personnel at the FIEB conservation breeding center.

### 2.2. Health Assessment and Physical Examination

Prior to sampling, animals were transported to the veterinary clinic in their own nest box, routinely used as a resting site within their enclosure. Upon arrival, they were transferred to a familiar handling cage used for regular fortnightly weight measurements. The handling cage was then placed inside a pre-oxygenated induction chamber and anesthesia was induced with isoflurane. Once an adequate anesthetic plane was achieved, animals were removed from the chamber, positioned on the examination table, and anesthesia was maintained via face mask throughout the procedure. A complete physical examination was carried out under isoflurane anesthesia as part of the annual health check-up program to confirm the absence of clinical disease. Physiological parameters including body temperature, heart and respiratory rates, snout-to-vent length, dental condition (including canine wear), body condition score (BCS) on a 5-point scale [29], body weight, and testicular size in males were recorded. Abdominal ultrasonography was also performed to assess kidney size and morphology.

Only animals with normal physiological parameters and no clinical signs of disease were considered eligible for the study. Because animals were monitored annually and some remained in the center for several years without being released, multiple samples were obtained from these individuals. In total, 75 individuals (37 females and 38 males) met the inclusion criteria and were incorporated into the study. Additional data—including age, sex, BCS, and microchip identification number—were recorded and available for the animals in the study.

### 2.3. Sample Collection

Blood samples were collected under isoflurane inhalation anesthesia within three minutes of removal from the induction chamber. Approximately 2.5–3 mLs of blood were drawn from the cranial vena cava using a sterile 25G × 1″ or 23G × 1″ needle (Nipro^®^ Needle, Nipro Medical Europe, Mechelen, Belgium) attached to a sterile 3-mL syringe (Injekt^®^ Luer Solo, B. Braun, Melsungen, Germany). Each sample was divided into three aliquots: one containing EDTA for hematological analyses, and two without anticoagulant (Aquisel^®^, Barcelona, España)—one for serum biochemistry and the other for serum protein electrophoresis. Serum was separated within approximately 20 min after collection. Samples collected in EDTA tubes were carefully inverted several times immediately after collection to ensure adequate mixing with the anticoagulant and stored at 4 °C until processing. Additionally, blood smears (EDTA) were performed and stained with a Wright’s Giemsa stain (StainRITE^®^ Wright-Giemsa Stain Solution, Gentaur Spain, Madrid, Spain). Smears were then examined under a light microscope to assess sample quality, including appropriate cellular distribution and the absence of platelet aggregates or other artifacts that could compromise interpretation. In addition to the health status that was previously determined, only samples meeting predefined quality criteria—namely absence of hemolysis, lipemia, clotting, or platelet aggregation—were included.

Between 2019 and 2025, 110 blood samples that met the study’s inclusion criteria were collected. Of these, 59 corresponded to males and 51 to females. Sampling frequency varied among individuals: 45 animals were sampled once, 25 were sampled twice, and 5 were sampled three times. Samples from the same animal were only included when collected in different years. Based on age at the time of sampling, 75 samples were obtained from juveniles and 35 from adults.

### 2.4. Laboratory Analyses

Blood samples for hematology and serum biochemistry were analyzed within 24 h from collection at an accredited veterinary diagnostic laboratory (Laboklin España, Alcobendas, Madrid, Spain), following standardized clinical pathology procedures. All pre-analytical steps, including sample collection, handling, and initial quality assessment, were performed by the research team.

A complete blood count and erythrocyte indices were obtained with an XN-1000V™ automated hematology analyser (Sysmex Spain, Sant Just Desvern, Barcelona, Spain) with multi-species software configured for ferrets. The evaluated hematological parameters included total red blood cell (RBC) count, hemoglobin concentration, hematocrit, erythrocyte indices such as mean corpuscular volume (MCV), mean corpuscular hemoglobin (MCH), and mean corpuscular hemoglobin concentration (MCHC), platelet count, and leukocyte variables, including total white blood cell (WBC) count and absolute and relative counts of neutrophils, lymphocytes, monocytes, eosinophils, and basophils.

Serum biochemical analytes were measured using a COBAS INTEGRA 400 plus™ automated biochemistry analyzer (Roche Diagnostics S.L., Sant Cugat del Vallès, Barcelona, Spain). The biochemical profile included energy and metabolic substrates (glucose, triglycerides, and cholesterol), renal parameters (urea and creatinine), protein profile variables (total protein, albumin, globulin, and albumin-to-globulin ratio [A:G]), hepatic and muscle enzymes (ALT, AST, ALP, GGT, GLDH, CK, amylase, and lipase), electrolytes and minerals (sodium, potassium, chloride, calcium, inorganic phosphate, magnesium, and iron), as well as vitamin E.

Hematological and biochemical results were interpreted based on samples obtained from clinically healthy animals, as determined by physical examination and ancillary testing, and were used for the establishment of reference intervals.

All blood parameters were expressed using International System of Units, except for enzyme activities, which were reported in units per liter (U/L).

Serological assays were conducted to detect antibodies against Severe Acute Respiratory Syndrome Coronavirus type 2 (SARS-CoV-2), Influenza A virus, *Leishmania infantum*, *Toxoplasma gondii*, and *Dirofilaria immitis*, following the methodologies described by Giner et al. [30] and Villanueva-Saz et al. [31,32,33]. All individuals tested seronegative. In addition, molecular testing for canine distemper virus (CDV) was performed in a subset of individuals using PCR assays as part of the health surveillance program routine, resulting negative in all cases. Although systematic screening was not performed in all the individuals during the study period, no clinical signs compatible or suggestive of CDV infection were observed in the colony under long-term veterinary monitoring.

### 2.5. Statistical Study

Statistical analyses were performed using SPSS version 31.0 (IBM Corp., Armonk, NY, USA, 2025). Reference intervals (RIs) were calculated using Reference Value Advisor v2.1 [34], following the recommendations of the International Federation of Clinical Chemistry and Laboratory Medicine (IFCC) and the Clinical and Laboratory Standards Institute (CLSI) guidelines [35] for the establishment of population-based reference intervals, particularly in small or heterogeneous datasets commonly encountered in wildlife and endangered species studies, as reported in previous wildlife studies using Reference Value Advisor [36,37,38].

Prior to RI estimation, descriptive statistics and graphical assessments (histograms, boxplots, and normal probability plots) were generated for each variable to evaluate data distribution and identify potential extreme values. Outliers were screened using both Tukey and Dixon procedures. In accordance with IFCC-CLSI recommendations [35], all detected outliers were retained in the dataset to preserve the natural biological variability of the study population, as none were associated with plausible analytical or biological artefacts (e.g., sample degradation, analytical error, or clinical abnormality), and their presence has been explicitly specified in the corresponding tables.

Normality of data distribution was evaluated using graphical inspection and formal statistical tests. Most hematological and biochemical variables displayed non-Gaussian or skewed distributions, which is common in wildlife health datasets due to variability in age structure, physiological condition, environmental exposure, and management background. Box–Cox transformation was automatically applied by the Reference Value Advisor, for improvement of distribution symmetry and variance stabilization prior to reference interval estimation [34].

Reference intervals were calculated according to sample size. For analytes with very small sample sizes (*n* < 20), the robust method combined with Box–Cox transformation was used; however, confidence intervals for the limits could not be reliably estimated due to limited statistical power, and these reference intervals should be interpreted as preliminary estimates. For analytes with sample sizes between 20 and 39, reference interval was also estimated using the robust method (with Box–Cox if appropriate), and the confidence intervals of the lower and upper limits were determined using a bootstrap approach to reflect the uncertainty associated with sampling in small populations. For analytes with sample sizes ≥ 40, reference intervals were calculated using the standard non-parametric (percentile-based) method, and the confidence intervals of the lower and upper limits were similarly determined using bootstrap resampling [34,35].

For all parameters, both lower and upper reference limits were reported. In addition to reference intervals, descriptive statistics including mean, median, standard error of the mean (SEM), minimum, maximum, and the 10th and 90th percentiles were calculated for each variable.

Differences between males and females, age groups (juveniles: 8 months–1 year, and adults > 1 year), and between animals with normal body condition (BCS = 3) and overweight (BCS = 4) were analyzed using the non-parametric Mann–Whitney test. These analyses were considered exploratory and were performed to identify potential sources of biological variation that might justify the partitioning of reference intervals. Therefore, *p* values were interpreted in a descriptive and exploratory manner and were not used for formal hypothesis testing, and no formal adjustment for multiple comparisons was applied. Statistical significance was set at *p* < 0.05. For analytes showing statistically significant differences between sexes or age groups (assessed by Mann– Whitney tests), separate reference intervals were also calculated. This approach allows the identification of biologically meaningful variation while maintaining sufficient sample size to ensure the reliability of the intervals, in accordance with IFCC-CLSI and ASVCP recommendations [35].

## 3. Results

### 3.1. Animals and Samples

A total of 110 samples from 75 clinically healthy European mink were included in the final dataset used to establish hematological and serum biochemical RIs. Selected mink fulfilled the predefined inclusion criteria regarding clinical examination, hematology and chemistry values, and negative pathogen serology screening. Inclusion criteria required: 37.5–39 °C rectal temperature immediately after anesthetic induction; absence of dehydration; a BCS between 2 and 4 (inclusive); pink or pale pink, moist mucous membranes; and no other evident clinical signs of disease (e.g., dermatitis or purulent nasal discharge). Blood quality was assessed in every sample, and samples with evidence of hemolysis, lipemia and/or inadequate cell distribution or quality of blood smears, were discarded from the study. Animals with a score of 2 or less in body condition were excluded from the analyses assessing the influence of body condition on hematological and biochemical values. Because blood samples were collected during routine health examinations and sample volume was limited, not all hematological and biochemical parameters could be determined for every individual and also sample quantity differed among analytes. The sample quantity for each analyte is specified in the corresponding tables.

### 3.2. Hematology

#### 3.2.1. Proposed Reference Intervals for Hematology

In Table 1, the mean, standard error of the mean (SEM), median, minimum, maximum, as well as the calculated RIs and their corresponding 90% CIs, for RBC count, hemoglobin concentration, hematocrit, erythrocyte indices (MCV, MCH, and MCHC), platelet count, and leukocyte variables—including WBC count and absolute and relative counts of neutrophils, lymphocytes, monocytes, eosinophils, and basophils—are shown. The Supplementary Material includes a more detailed table containing all calculated descriptive statistics, as well as the outliers identified and retained for each analyte (Table S1).

#### 3.2.2. Sex-, Age- and Body Condition-Related Differences in Hematology

Several hematological parameters showed significant differences for sex, age, and body condition in the European mink. Mean ± SEM values for all hematological parameters by category are shown in Table S3.

Males exhibited significantly higher hemoglobin concentrations compared to females (*p* = 0.031), while females showed higher platelet counts (*p* = 0.014) and total WBCs counts (*p* = 0.002). This increase in WBCs in females was mainly driven by significantly higher absolute (*p* = 0.003) and relative (*p* = 0.004) lymphocyte values, whereas males presented higher neutrophil (*p* = 0.008) and monocyte (*p* = 0.009) percentages, resulting in a higher neutrophil-to-lymphocyte profile. Hence, the mean, SEM, median, minimum, maximum, the 10th and 90th percentiles and RIs of these analytes were calculated separately for males and females (Table S4B).

Age-related differences were less pronounced for the erythrogram variables but were evident in leukocyte profiles. Juvenile mink showed significantly higher total WBC (*p* = 0.047) and lymphocyte (*p* = 0.038) counts compared to the adults, while adults displayed higher monocyte (*p* = 0.011) percentages. The mean, SEM, median, minimum, maximum, the 10th and 90th percentiles and RIs of these analytes were also calculated separately for juveniles and adults (Table S4C).

Body condition influenced mainly red cell parameters, with overweight individuals showing significantly higher hemoglobin concentration (*p* = 0.018) and hematocrit values (*p* = 0.005) than mink with normal body condition. No significant differences in leukocyte counts or differential percentages were observed between body condition categories.

### 3.3. Serum Biochemistry

#### 3.3.1. Proposed Reference Intervals for Serum Biochemistry

For serum biochemistry, RIs were generated for energy and metabolic analytes (glucose, triglycerides, and fructosamine); protein profile variables (total protein, albumin, globulin fractions, and the A:G ratio); hepatic enzymes (ALT, AST, ALP, GGT, and GLDH); muscle integrity marker (CK); renal parameters (urea and creatinine); minerals and electrolytes (sodium, potassium, chloride, calcium, phosphate, magnesium and iron); and vitamin E. The mean, SEM, median, minimum, maximum, as well as the calculated RIs and their corresponding 90% CIs, are shown in Table 2.

For analytes with limited sample size (*n* < 20; glucose, triglycerides, and chloride), the reported interval estimates should be considered preliminary and interpreted with caution due to the reduced statistical power associated with these datasets. A more detailed table containing all calculated descriptive statistics, together with the outliers identified and retained for each analyte, is provided in the Supplementary Material (Table S2).

#### 3.3.2. Sex-, Age- and Body Condition-Related Differences in Serum Biochemistry

Significant differences in several biochemical parameters were observed according to sex, age, and body condition in the European mink. Table S5 presents the mean ± SEM values for all biochemical parameters by category.

Variations based on sex were particularly evident in protein metabolism, lipid profile, renal markers, and several enzymes. Females had significantly higher cholesterol concentrations (*p* = 0.001), ALP (*p* = 0.002), urea (*p* < 0.001), total globulins (*p* = 0.005), and lower A:G ratios (*p* < 0.001) compared to males. In contrast, males exhibited significantly higher fructosamine (*p* = 0.011), ALT (*p* < 0.001), creatinine (*p* < 0.001), potassium (*p* < 0.001), phosphate (*p* = 0.047), and sodium (*p* = 0.016) concentrations. The mean, SEM, median, minimum, maximum, the 10% and 90% percentiles and RIs of these analytes were also calculated separately for males and females (Table S6).

Age-related biochemical differences were notable in protein fractions and enzyme activities. Adults had significantly higher globulin (*p* < 0.001) and lower A:G ratios (*p* < 0.001) compared with juveniles, indicating age-associated changes in immune protein production. Juveniles showed higher ALP activity (*p* = 0.016) and calcium concentrations (*p* = 0.015), consistent with skeletal growth and bone metabolism. Creatinine concentrations were also significantly higher (*p* = 0.014) in adults. The mean, SEM, median, minimum, maximum, the 10% and 90% percentiles and RIs were additionally calculated separately for juveniles and adults (Table S7).

Body condition effects were limited but included significantly higher sodium (*p* = 0.045) and magnesium (*p* = 0.044) concentrations in overweight mink. In contrast, most metabolic and enzymatic parameters did not differ between body condition groups, with cholesterol being the only analyte showing significantly lower concentrations (*p* = 0.034) in overweight mink.

## 4. Discussion

This study provides the first comprehensive listing of hematological and biochemical parameter reference intervals in the European mink. The establishment of reliable RIs for this species is essential for accurate clinical interpretation, health surveillance, and conservation management. These intervals are often unavailable for inaccessible or critically endangered species with small surviving cohorts, such as the European mink, and thus health personnel must rely on reference intervals extrapolated from related species. Reference intervals from related mustelids such as the ferret or the American mink are commonly used for the European mink. However, the accuracy of the extrapolation is unknown or questionable.

Overall, the hematological and biochemical values in this study are biologically plausible and consistent with previously reported values in American mink and domestic ferrets [22,23,24]. The relatively narrow dispersion of most analytes denotes the controlled management conditions of the breeding colony and the strict inclusion of clinically healthy individuals, thus highlighting the robustness of these reference intervals for clinical application in captive populations.

All hematological analyses were performed using a ferret-configured analyzer due to the current lack of species-specific calibration for European mink. Given the close taxonomic relationship between species within the genus Mustela and their comparable hematological characteristics, the standardized analytical approach applied to all the samples ensures internal consistency of the dataset and supports reliable comparisons within the studied population.

Certain limitations should be acknowledged when interpreting the results of this study. Repeated measurements from the same individual can introduce some degree of non-independence within values [39]; however, the number of repeated measurements per individual was limited (maximum of three), and sampling was performed under standardized conditions, which would have minimized the potential impact on the estimated reference intervals. Importantly, repeated samples included in the study were collected at least one year apart. The extended time interval between sample collections reduces the likelihood of dragging stable, individual-specific traits and increases the potential influence of external factors, thereby supporting the treatment of the samples as independent observations. Although repeated measurements cannot be considered fully independent and some residual within-individual correlation cannot be completely excluded, their limited number and broad temporal separation suggest that any resulting bias is likely to be small. In addition, the number of animals in this study was considered sufficient given the relatively small size of the entire population in Spain (approximately 142 individuals in the wild and 65 in captivity) [10]. According to the recommendations of the American Society for Veterinary Clinical Pathology [40], de novo reference intervals for veterinary species may be established using sample sizes greater than 20 individuals; hence, a population of 75 clinically well-characterized animals provides a solid foundation for the reference intervals obtained in this study.

Another limitation of the study was the unequal sample size across analytes, which resulted from the availability of serum volume and analytical constraints. Consequently, the precision of the estimated reference intervals and the statistical power of group comparisons may have varied among analytes.

In consonance with current guidelines, the reference intervals calculated for analytes with fewer than 20 samples (glucose, triglycerides, and chloride) should be interpreted as a guide rather than a fully reliable reference interval, and as such, considered as a preliminary estimate. However, given the absence of previous biochemical data for the European mink, these results provide valuable preliminary baseline information for future research.

Comparisons among sex, age, and body condition groups were intended to explore potential biological sources of variation rather than test specific a priori hypotheses. Consequently, statistically significant associations should be interpreted with caution and warrant confirmation in future studies.

A further limitation of this study was that sampling was performed exclusively outside of the breeding season. Given that the studied population was part of the ex situ conservation program for the European mink, expert panel guidelines and indications to avoid disturbances and human interactions that could hinder natural reproductive behavior were prioritized over sampling.

However, it is well established that reproductive physiological states may influence both biochemical and hematological parameters, particularly in females. Changes associated with estrus, pregnancy, and lactation can affect hematological profiles, including red cell indices during gestation due to hemodilution, as well as leukocyte profiles related to hormonal and metabolic modulation. In addition, biochemical parameters involved in energy metabolism (e.g., glucose and triglycerides), protein metabolism (e.g., total protein and albumin), and hepatic and lipid metabolism may also be affected by these physiological states, potentially leading to physiological variation in serum biochemical profiles [41]. Therefore, the reference intervals reported in this study should be interpreted as applicable to non-reproductive, clinically healthy individuals and may not be directly transferable to animals in active reproductive stages.

To facilitate the interpretation of the data discussed in this section, a comparison of the results obtained in this study and previously published data in the American mink and the domestic ferret is shown in Table 3. Despite the differences in methodology between our study and previously published work [20,21,22,23,24]—regarding species, age classes, anesthetic protocols, housing conditions, and statistical approaches—the scarcity of hematological and biochemical studies in mustelids makes these references valuable for contextual comparison. Many of the differences in study design and sampling conditions are inherently linked to the limited availability of samples from a critically endangered species where only few individuals remain. This contrasts highly with other farmed or domestic species, such as the American mink or the ferret. Given these limitations, the available datasets were considered useful for comparison, despite the methodological differences among studies. Notably, the values obtained in our study remained stable throughout the sampling periods, supporting the internal consistency and reliability of the dataset.

Anesthetic drugs such as isoflurane, used during sampling in our study, can induce mild physiological variations in hematological [42,43,44] and biochemical analytes [44]. All samples were collected under similar anesthetic conditions which ensured internal consistency and reliable comparability of the results. European mink should not be manually restrained for blood collection according to the current European husbandry guidelines [28]. These state that manual handling should be avoided whenever possible, making chemical immobilization the only welfare-compliant approach for sampling this species. Isoflurane was the drug of choice in this study. While the 1996 guidelines [28] recommended injectable anesthesia—mainly because isoflurane was not widely accessible at that time—the updated European guidelines that are currently being developed by experts in the field (unpublished data) identify isoflurane as the preferred anesthetic agent for European mink due to its safety profile, rapid recovery, and minimal physiological impact.

Hematological parameters, such as mean RBC count, hemoglobin concentration, and hematocrit values in European mink were similar to the parameters in captive American mink populations and fell within the broad reference intervals for ferrets, despite notable interspecific and methodological differences among studies (Table 3A). Median values were slightly shifted to the left, when compared to studies in ferrets that had not been anesthetized [22]. Higher RBC counts due to cortisol-induced splenic contraction are expected with venipuncture without sedation [41], which could explain the left shift variation in values under isoflurane anesthesia.

The erythrocyte index MCHC in European mink was in between the values reported for American mink and ferrets, but both American mink and ferrets tended to show lower mean MCV values. These patterns likely display species-specific hematological traits more than pathological differences.

In this study, platelet counts exhibited higher dispersion. Compared to previous studies, platelet counts in European mink were lower than those reported in American mink studies but were well within the ranges described for ferrets. Considerable variability in platelet values across studies has been previously attributed to differences in analytical methods, sample handling, species-specific platelet aggregation tendencies and method of anesthesia [23,24], which may in part explain the discrepancies.

Total WBC counts and leukocyte differentials in European mink closely overlapped with values reported for both American mink and ferrets. Monocyte and lymphocyte counts were particularly similar to those described in anesthetized American mink and fell within the published reference intervals for domestic ferrets. Minor differences observed for eosinophils and basophils are unlikely to be clinically relevant and may reflect differences in environmental exposure, husbandry conditions, or immune responses among study populations. However, our dataset revealed a comparatively wider dispersion in total leukocyte and neutrophil counts. Similar variability has been described in captive and semi-free-ranging mustelids [24] and may suggest differences in handling, environmental exposure, or physiological stress response associated with management and translocation rather than underlying disease. Importantly, animals showing leukocyte values outside internal screening thresholds or alterations in blood smears were excluded from the RI, to further ensure that these intervals represent health values for the species.

Importantly, comparisons between studies should be interpreted with caution given that factors such as age distribution, anesthesia protocols, housing conditions, and analytical platforms varied substantially. In particular, the use of anesthesia in the present work and in several American mink studies may have influenced selected hematological parameters, including hematocrit and leukocyte counts, and could explain the differences in these parameters in unanesthetized ferrets [24].

Biochemical analytes also revealed similarities and notable differences between the American mink and the ferret (Table 3B). Serum glucose concentrations in European mink were higher on average than those reported for American mink and domestic ferrets. The reference interval range was wider in European mink than in general for the American mink and the ferret, possibly due to the variations in parameters caused by the anesthetic protocols, handling-related stress, or fasting status [41]. Stress-induced hyperglycemia has been well documented in a variety of carnivore species, including mustelids [17,41], although the magnitude of this response may vary depending on species and management conditions. This phenomenon is particularly relevant under anesthesia or captivity and is therefore a plausible explanation for observed discrepancies rather than an indicator of underlying metabolic disease. In contrast, fructosamine concentrations in European mink were lower than those reported in ferrets. Given that fructosamine reflects average glycemic regulation over several weeks, this marker is less influenced by acute stress or immediate pre-analytical variation and therefore provides a complementary assessment of longer-term glucose metabolism. These differences may reflect species-specific protein turnover rates, albumin concentrations, or dietary carbohydrate intake [17]. Methodological variation and differences in age distribution between studies may also have contributed to these discrepancies.

Triglyceride concentrations in European mink were lower than those reported for American mink and fell within the lower range of values in ferrets. These differences may be attributable to diet, differences in lipid metabolism among species, or age-related factors [20,21,23], or methodological variation among studies, particularly given that some reports were based exclusively on juvenile cohorts [21,23]. It should also be noted that triglyceride results in this study were based on a limited number of samples (*n* = 8), and therefore should be interpreted as preliminary estimates. Cholesterol concentration values in European mink fell between the values reported for American mink and ferrets, supporting a conserved lipid metabolic profile among mustelids [23,24], with interspecific variation likely due to diet and husbandry rather than pathology. It is also important to consider seasonal fluctuations at the time of sample collection. Potential fluctuations during breeding season have been reported in American mink [45]—particularly affecting proteins, triglycerides, and cholesterol. Such fluctuations are unlikely to affect our dataset, given that the adult European mink were sampled during the non-breeding season (August–February) [26]. The stability of the values throughout sampling further supports the internal consistency of the results and strengthens the reliability of the reference intervals.

Albumin, globulin fractions, and the A:G ratio were consistent with previously published data from our group, establishing healthy immunoglobulin parameters in the European mink [25]. Minor differences were noted, plausibly due to the larger sample size of the present study. Total protein and albumin concentrations in European mink were lower than those reported for American mink and ferrets. These results may reflect a combination of physiological differences among species and differences in husbandry, nutrition, and management conditions between study populations. Overall, the protein profile was consistent with an immunologically healthy population. The observed discrepancies with previously published data reinforce the importance of establishing dedicated RIs for European mink [17,20,25].

Alanine aminotransferase and AST values in European mink were lower than those reported in juvenile American mink but overlapped with values in ferrets. Elevated aminotransferase in American mink kits likely indicates age-related metabolic activity and growth [17,21,41] rather than hepatic disease. Differences among studies may also be influenced by muscle activity, anesthesia, and analytical platforms. Importantly, although ferrets are a domesticated species in which stress-related variation is expected to be reduced, published reference intervals for ALT and AST remain relatively wide, suggesting that a substantial component of variability is intrinsic to these enzymes rather than solely attributable to handling or capture effects. Alkaline phosphatase activity in European mink was markedly lower than that reported in juvenile American mink but similar to values described in adult ferrets. This pattern was consistent with age-associated bone metabolism given that higher ALP activity is expected in growing animals [17,23]. The inclusion of both adult and juvenile cohorts in the present study likely contributed to the wider reference intervals. Gamma-glutamyl transferase and GLDH were low and comparable among species, supporting the absence of clinically relevant hepatobiliary disease in European mink. Minor differences between studies are likely attributable to methodological variability rather than biological divergence.

Creatine kinase activity in European mink was substantially lower than values reported in American mink and within the lower range described in ferrets. Creatine kinase is highly sensitive to muscle handling, restraint, and intramuscular injections [17,41,46]. However, the observation that relatively wide CK reference intervals are also reported in ferrets further suggests that variability in this enzyme is not exclusively related to capture or handling stress, but also reflects inherent physiological variation in muscle metabolism. Therefore, the lower CK values observed in European mink compared to American mink or domestic ferrets may indicate standardized sampling procedures and careful handling during anesthesia. In wildlife species, the wide reference intervals commonly reported for CK are generally associated with increased muscular activity and/or muscle damage [47].

Amylase and lipase values in European mink were lower than those reported for American mink and markedly lower than those in ferrets. These differences may reflect a combination of physiological variation among species and differences in husbandry, nutrition, and analytical methodologies across study populations [41]. In particular, differences in diet composition and feeding management among captive mustelid populations may contribute to variation in digestive enzyme activity. No concurrent biochemical or clinical findings indicative of pancreatic disease were observed in the animals studied; therefore, the results were interpreted as physiological.

Renal markers showed relatively tight intervals, suggesting good physiological homogeneity for creatinine and urea in clinically healthy individuals maintained under standardized husbandry conditions. Urea and creatinine concentrations in European mink were lower than those reported for American mink but overlapped with values in ferrets. Lower creatinine concentrations may be associated with differences in body mass, muscle mass, or dietary protein intake [17,23,41]. Given the absence of concurrent electrolyte abnormalities or clinical signs, these differences were interpreted as physiological rather than indicative of renal dysfunction.

Electrolyte concentrations (sodium, potassium, and chloride) in European mink closely matched those reported for American mink and ferrets, indicating conserved electrolyte homeostasis among mustelids. Slightly higher potassium concentrations reported in juvenile American mink likely suggest age-related cellular turnover or hemolysis. Calcium and phosphate concentrations in European mink were within the ranges reported for ferrets but lower than those described for juvenile American mink, again supporting an age-related explanation linked to skeletal growth. Magnesium concentrations were lower in European mink than in the other mustelids, maybe indicating differences in diet, element absorption and metabolism, or other husbandry-related factors. Iron concentrations were comparable to those reported in ferrets, supporting an adequate iron status of the population and reinforcing the lack of chronic disease or inflammation [17,23,41].

Overall, European mink shared broadly similar hematological and biochemical profiles with other mustelids; nevertheless, the differences observed among studies reinforce the need for reliable reference intervals for European mink, particularly in conservation medicine.

In the analyses based on sex, higher hemoglobin concentrations were observed in male European mink, consistent with findings in American mink [48] and domestic ferrets [49]. Males typically present higher red cell mass due to androgen-mediated stimulation of erythropoiesis. Females showed higher total leukocyte counts, lymphocyte counts and percentages, and lower neutrophil and monocyte percentages. Female American mink show higher lymphocyte proportions and lower neutrophil dominance compared with males [50]. This result may indicate sex-related differences in immunity. In mammals, females often exhibit a more pronounced adaptive immune profile, characterized by higher lymphocyte numbers, whereas males tend to show relatively higher innate immunity cell counts, partly influenced by the immunomodulatory effects of sex hormones and response to stress [51,52]. Similar sex-associated leukocyte patterns have been reported in other carnivores and are generally considered physiological variations instead of pathological when animals are clinically healthy [17,23]. The higher platelet values observed in females are also in agreement with previous studies in mustelids [20,23].

Sex-based differences in lipid and protein metabolism in European mink mirror those described in ferrets [23], American [50] and European mink [25]. Elevated cholesterol and globulin concentrations in females have been linked to reproductive physiology and estrogen-mediated modulation of lipid transport and immune protein synthesis [23]. The lower A:G ratio observed in females further supports increased globulin production, a pattern also reported in female American mink [25,50]. Higher creatinine concentrations in males is widely documented among mustelid species and is generally attributed to greater muscle mass rather than renal dysfunction. Similar patterns have been reported in American mink [50] and ferrets [23], where sex-associated differences in muscle-derived metabolites are considered physiologically normal. Mild differences according to sex have been reported for liver enzymes in mustelids, particularly ALT [23,50]. In agreement with the present study, males showed higher ALT values than females in previous reports. Such differences may be associated with sex-related variation in hepatic metabolism or muscle enzyme activity rather than indicating true hepatocellular injury.

These findings are consistent with sex-based immunological and metabolic differences described in mustelids and other mammals, often linked to hormonal influence and differences in body composition. These results highlight the importance of sex-specific interpretation when evaluating biochemical profiles in European mink, especially in conservation and breeding programs.

Age-related leukocyte variations in European mink, particularly the higher lymphocyte counts in juveniles, are consistent with similar immune system maturation patterns in ferrets. Juvenile mustelids typically exhibit a lymphocyte-predominant leukogram, while adults develop a more balanced leukocyte distribution as immunological memory increases [23,24].

The significantly higher globulin concentration in adult European mink is in agreement with the previous studies in European mink [25], indicating cumulative antigenic exposure and immunoglobulin production over time. A decrease in the A:G ratio has been consistently reported in adult mustelids [23,25] and is considered a normal age-related finding. Therefore, the age-related variations observed in the protein profile can be considered physiological given that adult animals typically exhibit higher globulin concentrations [18]. Higher ALP and AST activities and calcium concentrations in juveniles are well documented in growing ferrets [23] and indicate active bone growth and osteoblastic activity [17,41]. These findings should be interpreted as physiological rather than pathological when evaluating young individuals. Lower vitamin E concentrations in juvenile mink compared to adults may be explained by the higher physiological demands associated with rapid growth and development. During early stages of life, increased cellular proliferation and elevated oxidative metabolism lead to a greater use of vitamin E as a lipid-soluble antioxidant, while the capacity for intestinal absorption and tissue storage of fat-soluble vitamins is not yet fully developed. In contrast, adult mink have more stable metabolic rates and more body reserves, allowing for higher and more stable vitamin E levels [53,54].

Although exploratory analyses suggested subtle sex- and age-related tendencies, separate reference intervals were calculated only for those analytes showing statistically significant differences among groups. A single species-wide reference interval was determined for all other parameters, appropriate for most clinical and conservation interventions. Nevertheless, bloodwork and biochemistry results should be interpreted with caution when using these intervals, particularly for juveniles and during breeding season, when certain physiological variations in analytes are expected [17].

Body condition appears to exert a modest but biologically meaningful influence on several analytical parameters in European mink. While leukocyte profiles were largely unaffected, suggesting that moderate variation in body reserves does not substantially alter baseline immune cell distributions, differences observed in erythrocytic parameters indicate a potential physiological adjustment associated with body size and metabolic demand. Higher hemoglobin and hematocrit values in overweight individuals may indicate increased oxygen transport capacity and circulating red cell mass, a pattern also described in other carnivores [55]. Similarly, body condition-related variation in electrolyte concentrations, including sodium and magnesium, may be linked to differences in hydration status, or metabolic regulation rather than body condition per se [18]. Given the scarcity of data addressing the relationship between body condition and hematological or biochemical profiles in wild and captive mustelids, these findings provide valuable baseline information and underscore the importance of accounting for body condition when interpreting analytical results in ecological, clinical, and conservation contexts.

## 5. Conclusions

This study provides the first comprehensive hematological and serum biochemical dataset for captive European mink, integrating the effects of sex, age, and body condition within a conservation-focused framework. Although reference intervals could not be established for a small number of analytes due to limited sample size, the descriptive statistics reported here offer valuable baseline information for future comparisons, particularly given the absence of prior species-specific data in the literature. Overall, the analytical profiles of European mink were broadly comparable to those of other mustelids, while still revealing consistent species-specific patterns and physiological modifiers that are essential for accurate clinical interpretation. In addition, European mink included in this study were maintained under conservation breeding conditions designed to preserve natural behaviors and minimize adaptation to captivity. Therefore, comparisons with domestic ferrets or farmed American mink should be interpreted within the context of these substantial differences in management objectives and husbandry practices.

Sex-related differences in erythrocytic, leukocyte, protein, lipid, and renal-associated parameters largely revealed the expected influence of hormones, immune status, and body composition, whereas age-related variation corresponded to normal processes of growth and immune development. Body condition exerted a modest but biologically meaningful effect on selected variables, underscoring the importance of considering nutritional and metabolic status when interpreting laboratory results from managed populations.

Although exploratory analyses identified certain tendencies associated with sex and age, the absence of strong partitioning effects supports the use of unified species-specific reference intervals for most routine clinical assessments. Nevertheless, a cautious interpretation of results remains advisable for juveniles and during breeding season, when physiological modulation of several analytes is expected.

Importantly, these reference values—derived from a single but demographically well-characterized captive population that represents nearly one-third of the remaining global stock—constitute a robust and highly relevant tool for conservation medicine. In this context, their clinical interpretation should always consider the standardized conditions of captive management and the inherent physiological variability associated with routine handling, anesthesia, and individual biological differences. Accordingly, these reference intervals should be regarded as applicable to clinically healthy captive European mink and should not be directly extrapolated to free-ranging populations, diseased individuals, or animals sampled under different handling protocols. The provided interpretative framework will facilitate standardized health monitoring, clinical decision-making, and welfare assessment in captive breeding, translocation, and reintroduction programs, ultimately supporting evidence-based management strategies for the long-term recovery of this critically endangered species.

## Figures and Tables

**Table 1:** Hematological analytes of a single captive collection of European mink (*Mustela lutreola*) in Spain. For all analytes, the mean, standard error of the mean, median, minimum, maximum, as well as the lower and upper reference intervals, including their 90% confidence intervals, were calculated.

Analyte (Unit)	*n*	Mean	SEM	Median	Minimum	Maximum	RI	LRL	URL
RBC (×10^12^/L)	97	7.89	0.08	8.05	4.24	9.19	5.9–9	4.2–6.5	8.8–9.2
Hemoglobin (g/L)	97	155.27	1.69	159.00	80.00	181.00	92.2–177.7	80–130.5	172.6–181.0
Hematocrit (%)	97	51.20	0.61	52.00	28.00	63.00	33.9–60.6	28–41.5	58.6–63
MCV (fL)	54	66.63	0.96	66.40	49.80	79.60	50.1–79.4	49.8–53.3	78.3–79.6
MCH (pg)	54	19.74	0.24	19.85	9.90	22.60	12.2–22.4	9.9–17.8	21.4–22.6
MCHC (g/L)	54	301.04	3.38	298.50	267.00	379.00	267–377.9	267–271.4	356.6–379.0
Platelets (×10^9^/L)	84	513.12	15.41	520.00	65.00	883.00	103.1–773.1	65.0–271.9	740.0–883.0
WBC (×10^9^/L)	97	4.97	0.24	4.70	1.20	15.80	1.6–11.2	1.2–1.9	8.8–15.8
Neutrophils (×10^9^/L)	97	1.96	0.26	1.40	0.50	23.00	0.6–10.2	0.5–0.7	4.2–23.0
Lymphocytes (×10^9^/L)	97	2.78	0.15	2.40	0.60	7.80	0.6–6.6	0.6–1.0	5.3–7.8
Monocytes (×10^9^/L)	97	0.19	0.01	0.20	0.00	.80	0.0–0.6	0.0–0.0	0.5–0.8
Eosinophils (×10^9^/L)	97	0.28	0.04	0.20	0.00	3.20	0.0–1.1	0.0–0.0	0.7–3.2
Basophils (×10^9^/L)	97	0.00	0.00	0.00	0.00	0.00	0.0–0.0	0.0–0.0	0.0–0.0
Neutrophils (%)	97	35.43	1.32	34.00	6.00	82.00	14.4–73.3	6.0–17.0	56.0–82
Lymphocytes (%)	97	55.13	1.36	57.00	13.00	81.00	13.5–77.7	13.0–32.4	73.6–81.0
Monocytes (%)	97	4.02	0.25	4.00	0.00	20.00	0.0–8.6	0.0–1.0	7.6–20.0
Eosinophils (%)	97	5.12	0.35	5.00	0.00	15.00	0.0–13.7	0.0–0.0	11.0–15.0
Basophils (%)	97	0.03	0.02	0.00	0.00	1.00	0.0–1.0	0.0–0.0	0.0–1.0

SEM: Standard error of the mean. RI: Reference interval. LRL: 90% confidence interval of the lower reference limit. URL: 90% confidence interval of the upper reference limit. MCV: Mean corpuscular volume. MCH: Mean corpuscular hemoglobin. MCHC: Mean corpuscular hemoglobin concentration. WBC: Total white blood cell count.

**Table 2:** Serum biochemical analytes of a single captive collection of European mink (*Mustela lutreola*) in Spain. For all analytes, the mean, standard error of the mean, median, minimum and maximum, were calculated. Lower and upper reference interval limits including 90% confidence intervals, are shown where applicable. For analytes with small sample size (*n* < 20), these interval estimates should be interpreted as preliminary due to limited statistical power (‡).

Analyte (Unit)	*n*	Mean	SEM	Median	Minimum	Maximum	RI	LRL	URL
Glucose (mmol/L)	7	8.23	1.01	8.55	4.90	12.98	3.4–17.5 ‡		
Triglycerides (mmol/L)	8	0.62	0.06	0.58	0.34	0.88	0.2–1.1 ‡		
Cholesterol (mmol/L)	100	5.17	0.08	5.10	3.40	7.70	3.8–7.1	3.4–4.0	6.6–7.0
Fructosamine (μmol/L)	96	120.71	3.90	113.5	82.00	366.00	87–268.6	82.0–89.0	155.0–366.0
Total protein (g/L)	104	52.28	0.39	52.00	43.00	62.00	45.6–60.4	43.0–46.6	60.0–62.0
Albumin (g/L)	104	28.52	0.31	28.50	19.00	38.00	23.0–36.8	19.0–24.0	33.4–38.0
Globulin (g/L)	104	23.76	0.34	23.00	17.00	35.00	18.0–31.0	17.0–18.6	30.0–35.0
A:G ratio	104	1.23	0.02	1.21	0.54	1.83	0.8–1.8	0.5–0.9	1.7–1.8
ALT (U/L)	109	121.35	6.64	101.0	46.00	555.00	53.5–313.5	46.0–63.0	230.5–555.0
AST (U/L)	108	55.26	4.65	38.50	19.00	431.00	20.0–164.9	19.0–23.5	106.4–431.0
ALP (U/L)	103	44.50	2.47	37.00	8.00	149.00	9.6–119.4	8.0–17.0	90.3–149.0
GGT (U/L)	86	4.79	0.73	3.00	0.00	42.00	0.00–26.7	0.0–0.0	17.7–42.0
GLDH (U/L)	52	0.88	0.10	0.70	0.00	3.20	0.00–3.2	0.0–0.1	2.4–3.2
Total bilirubin (μmol/L)	97	0.71	0.06	0.60	0.00	3.50	0.00–2.5	0.0–0.1	1.8–3.5
CK (U/L)	108	149.94	11.85	117.0	0.00	683.00	7.2–533.7	0.0–40.4	411.9–683.0
Amylase (U/L)	103	61.93	2.16	60.00	0.00	223.00	6.4–95.8	0.0–42.8	85.0–223.0
Lipase (U/L)	102	24.04	0.89	23.00	0.00	86.00	7.5–40.3	0.00–15.0	37.0–86.0
Urea (mmol/L)	109	10.88	0.33	10.30	5.70	22.30	5.9–20.1	5.7–6.4	17.0–22.3
Creatinine (μmol/L)	109	35.47	0.94	34.00	16.00	69.00	16.0–59.8	16.0–21.8	52.8–69.0
Sodium (mmol/L)	102	152.82	0.35	152.5	144.00	165.00	146.0–164.4	144.0–147.6	157.9–165.0
Potassium (mmol/L)	102	4.31	0.04	4.30	3.70	6.50	3.7–5.4	3.7–3.7	5.1–6.5
Chloride (mmol/L)	8	119.38	1.74	118.5	113.00	130.00	106.2–130.3 ‡		
Calcium (mmol/L)	103	2.15	0.02	2.20	1.50	2.50	1.8–2.4	1.5–1.9	2.4–2.5
Phosphate (mmol/L)	103	1.32	0.04	1.30	0.60	2.20	0.6–2.2	0.6–0.7	2.0–2.2
Magnesium (mmol/L)	100	0.82	0.01	0.80	0.40	1.10	0.6–1.0	0.4–0.7	1.0–1.1
Iron (mmol/L)	100	31.07	0.97	30.75	3.70	52.50	4.6–51.7	3.7–16.0	44.9–52.5
Vitamin E (μmol/L)	34	40.48	3.25	34.80	10.44	92.34	14.9–92.6	12.2–19.4	64.1–130.8

‡ Reference intervals should be interpreted with caution as preliminary estimates in these cases. SEM: Standard error of the mean. RI: Reference interval. LRL: 90% confidence interval of the lower reference limit. URL: 90% confidence interval of the upper reference limit. A:G ratio: Albumin–to–globulin ratio. ALT: Alanine aminotransferase. AST: Aspartate aminotransferase. ALP: Alkaline phosphatase. GGT: Gamma–glutamyl transferase. GLDH: Glutamate dehydrogenase. CK: Creatine kinase.

**Table 3:** Mean, median and reference interval for various blood analytes (A: Hematology, B: Biochemistry) in the present study compared with published values for American mink (*Neogale vison*) and domestic ferrets (*Mustela putorius furo*). (‡) Reference intervals should be interpreted cautiously as preliminary estimates in these cases. Literature values are included for comparison purposes. Table 3A is an adaptation of the table in Schalm’s et al. [24].

A: Hematology					
Analyte	Unit	Mean	Median	Reference Interval	Animal
RBC (×10^12^/L)	×10^12^/L	7.89	8.05	5.9–9	European mink ^a^
		7.90	-	-	American mink ^b^
		9.23	-	-	American mink ^c^
		-	10.5	7.4–13.0	Ferret ^d^
		-	-	4.8–7.8	Ferret ^e^
		-	-	6.6–12.2	Ferret ^f^
Hemoglobin (g/L)	g/L	155.27	159.00	92.2–177.7	European mink ^a^
		155.50	–	–	American mink ^b^
		157.20	–	–	American mink ^c^
		–	178.90	139.0–219.0	Ferret ^d^
		–	–	96.0–138.0	Ferret ^e^
		–	–	108.0–197.0	Ferret ^f^
Hematocrit (%)	%	51.20	52.00	33.9–60.6	European mink ^a^
		46.6	–	–	American mink ^b^
		49.0	–	–	American mink ^c^
		–	60.0	40–70	Ferret ^d^
		–	–	27–39	Ferret ^e^
		–	–	35–58	Ferret ^f^
MCV	fL	66.63	66.40	50.1–79.4	European mink ^a^
		59.05	–	–	American mink ^b^
		52.99	–	–	American mink ^c^
		–	54.3	49.6–60.6	Ferret ^d^
		–	–	47.8–57.6	Ferret ^e^
		–	–	42–60	Ferret ^f^
MCHC	g/L	301.04	298.50	267–377.9	European mink ^a^
		333.5	–	–	American mink ^b^
		323.0	–	–	American mink ^c^
		–	311.0	286.8–336.8	Ferret ^d^
		–	–	347.0–370.0	Ferret ^e^
		–	–	284.0–379.0	Ferret ^f^
Platelets	×10^9^/L	513.12	520.00	103.1–773.1	European mink ^a^
		785.14	–	–	American mink ^b^
		274.38	–	–	American mink ^c^
		–	807.0	171.7–1280.6	Ferret ^d^
		–	–	382–830	Ferret ^e^
		–	–	300–900	Ferret ^f^
WBC	×10^9^/L	4.97	4.70	1.6–11.2	European mink ^a^
		5.88	–	–	American mink ^b^
		5.22	–	–	American mink ^c^
		–	7.2	3.0–16.7	Ferret ^d^
		–	–	5.3–12.6	Ferret ^e^
		–	–	2.2–11.3	Ferret ^f^
Neutrophils	×10^9^/L	1.96	1.40	0.6–10.2	European mink ^a^
		2.48	–	–	American mink ^b^
		–	3.0	0.9–7.4	Ferret ^d^
		–	–	1.5–6.5	Ferret ^e^
		–	–	0.7–7.4	Ferret ^f^
Lymphocytes	×10^9^/L	2.78	2.40	0.6–6.6	European mink ^a^
		2.74	–	–	American mink ^b^
		–	3.40	0.6–10.5	Ferret ^d^
		–	–	1.8–8.6	Ferret ^e^
		–	–	0.5–5.6	Ferret ^f^
Monocytes	×10^9^/L	0.19	0.20	0.0–0.6	European mink ^a^
		0.185	–	–	American mink ^b^
		–	0.2	0–0.5	Ferret ^d^
		–	–	0.1–0.5	Ferret ^e^
		–	–	0.0–0.5	Ferret ^f^
Eosinophils	×10^9^/L	0.28	0.20	0.0–1.1	European mink ^a^
		0.425	–	–	American mink ^b^
		–	0.1	0–0.7	Ferret ^d^
		–	–	0.1–0.7	Ferret ^e^
		–	–	0.0–0.6	Ferret ^f^
Basophils	×10^9^/L	0.00	0.00	0.0–0.0	European mink ^a^
		0.04	–	–	American mink ^b^
		–	0.00	0.0–0.2	Ferret ^d^
		–	–	0.0–0.1	Ferret ^e^
		–	–	0.0–0.1	Ferret ^f^
**B** **: Biohemistry**					
**Analyte**	**Unit**	**Mean**	**Median**	**Reference Interval**	**Animal**
Glucose	mmol/L	8.23	8.55	3.4–17.5 ‡	European mink ^a^
		6.98	–	–	American mink ^b^ (M)
		6.88	–	–	American mink ^b^ (F)
		5.22	–	–	American mink ^c^
		–	6.0	3.0–8.5	Ferret ^d^
Triglycerides	mmol/L	0.62	0.58	0.2–1.1 ‡	European mink ^a^
		1.40	–	–	American mink ^c^
		–	1.0	0.5–2.8	Ferret ^d^
Cholesterol	mmol/L	5.17	5.10	3.8–7.1	European mink ^a^
		8.31	–	–	American mink ^c^
		–	4.9	2.4–7.1	Ferret ^d^
Fructosamine	μmol/L	120.71	113.50	87–268.6	European mink ^a^
		–	163.0	121.1–201.6	Ferret ^d^
Total protein	g/L	52.28	52.00	45.6–60.4	European mink ^a^
		59.4	–	–	American mink ^b^ (M)
		60.9	–	–	American mink ^b^ (F)
		75.53	–	–	American mink ^c^
		–	67.8	54.7–77.9	Ferret ^d^
Albumin	g/L	28.52	28.50	23.0–36.8	European mink ^a^
		29.8	–	–	American mink ^b^ (M)
		30.0	–	–	American mink ^b^ (F)
		36.21	–	–	American mink ^c^
		–	36.1	28.0–43.9	Ferret ^d^
ALT	U/L	121.35	101.00	53.5–313.5	European mink ^a^
		71.6	–	–	American mink ^b^ (M)
		80.0	–	–	American mink ^b^ (F)
		222.12	–	–	American mink ^c^
		–	110.0	49.0–242.8	Ferret ^d^
AST	U/L	55.26	38.50	20.0–164.9	European mink ^a^
		67.0	–	–	American mink ^b^ (M)
		76.3	–	–	American mink ^b^ (F)
		213.13	–	–	American mink ^c^
		–	74.0	40.1–142.7	Ferret ^d^
ALP	U/L	44.50	37.00	9.6–119.4	European mink ^a^
		135.89	–	–	American mink ^c^
		–	34.0	13.3–141.6	Ferret ^d^
GGT	U/L	4.79	3.00	0.00–26.7	European mink ^a^
		5.86	–	–	American mink ^c^
		–	4.0	0.2–14.0	Ferret ^d^
GLDH	U/L	0.88	0.70	0.00–3.2	European mink ^a^
		–	1.0	0.0–2.5	Ferret ^d^
Total bilirubin	μmol/L	0.71	0.60	0.00–2.5	European mink ^a^
		8.86	–	–	American mink ^c^
		–	1.1	0.0–3.3	Ferret ^d^
CK	U/L	149.94	117.00	7.2–533.7	European mink ^a^
		1091.51	–	–	American mink ^c^
		–	203.0	94.0–730.9	Ferret ^d^
Amylase	U/L	61.93	60.00	6.4–95.8	European mink ^a^
		112.66	–	–	American mink ^c^
		–	38.0	19.4–61.9	Ferret ^d^
Lipase	U/L	24.04	23.00	7.5–40.3	European mink ^a^
		–	204.0	73.2–351.1	Ferret ^d^
Urea	mmol/L	10.88	10.30	5.9–20.1	European mink ^a^
		5.43	–	–	American mink ^b^ (M)
		5.78	–	–	American mink ^b^ (F)
		3.36	–	–	American mink ^c^
		–	9.8	4.8–16.9	Ferret ^d^
Creatinine	μmol/L	35.47	34.00	16.0–59.8	European mink ^a^
		62.8	–	–	American mink ^b^ (M)
		55.7	–	–	American mink ^b^ (F)
		74.63	–	–	American mink ^c^
		–	44.0	23.0–76.7	Ferret ^d^
Sodium	mmol/L	152.82	152.50	146.0–164.4	European mink ^a^
		153.7	–	–	American mink ^b^ (M)
		153.4	–	–	American mink ^b^ (F)
		156.98	–	–	American mink ^c^
		–	154.0	140.1–169.7	Ferret ^d^
Potassium	mmol/L	4.31	4.30	3.7–5.4	European mink ^a^
		4.34	–	–	American mink ^b^ (M)
		4.51	–	–	American mink ^b^ (F)
		7.82	–	–	American mink ^c^
		–	5.0	3.9–5.9	Ferret ^d^
Chloride	mmol/L	119.38	118.50	106.2- 130.3 ‡	European mink ^a^
		114.5	–	–	American mink ^b^ (M)
		114.6	–	–	American mink ^b^ (F)
		112.76	–	–	American mink ^c^
		–	114.0	108.0–119.9	Ferret ^d^
Calcium	mmol/L	2.15	2.20	1.8–2.4	European mink ^a^
		9.54	–	–	American mink ^b^ (M)
		9.47	–	–	American mink ^b^ (F)
		1.19	–	–	American mink ^c^
		–	2.3	2.0–2.6	Ferret ^d^
Phosphate	mmol/L	1.32	1.30	0.6–2.2	European mink ^a^
		1.73	–	–	American mink ^b^ (M)
		1.69	–	–	American mink ^b^ (F)
		2.40	–	–	American mink ^c^
		–	1.8	1.0–3.1	Ferret ^d^
Magnesium	mmol/L	0.82	0.80	0.6–1.0	European mink ^a^
		1.47	–	–	American mink ^c^
		–	1.2	0.9–1.6	Ferret ^d^
Iron	mmol/L	31.07	30.75	4.6–51.7	European mink ^a^
		–	33.8	11.7–56.3	Ferret ^d^

^a^ Anesthetized, healthy, adult and juvenile, captive, European mink (*n* = 110), Statistics and RI generation done in accordance with ASVCP guidelines (Present study); ^b^ Anesthetized, healthy, adult, captive, American brown mink (*n* = 160) [20]. M: males; F: Females. The mean hematological values for both sexes were extracted from Clothier (2022) [24]; ^c^ Anesthetized, healthy, 6-month-old American black mink (*n* = 20) [21]; ^d^ Eleven weeks to 9-year-old, mixed sex, unanesthetized, healthy pet ferrets (*n* = 106), Statistics and RI generation done in accordance with ASVCP guidelines. Significant differences in age, sex, and fasted status were noted by the authors, but details were not provided [22]; ^e^ Domestic ferrets 10–12-week-old, mixed sex, anesthetized, using cardiac puncture [23]; ^f^ Adult, mixed sex, apparently healthy domestic ferrets (*n* > 500). Anesthetic status unknown; however, from the comparatively decreased hematocrit and WBC, it is possible that many of the ferrets were anesthetized, RI generated in accordance with ASVCP guidelines [24].

## Data Availability

The original contributions presented in this study are included in the article/Supplementary Material. Further inquiries can be directed to the corresponding author(s).

## Supplementary Materials

Supporting information can be downloaded at: https://www.mdpi.com/article/10.3390/vetsci13070619/s1.

## Abbreviations

The following abbreviations are used in this manuscript:

ALP	Alkaline phosphatase
ALT	Alanine aminotransferase
AST	Aspartate aminotransferase
A:G	Albumin-to-globulin ratio
BCS	Body condition score
CBC	Complete blood count
CDV	Canine distemper virus
CI	Confidence interval
CK	Creatine kinase
EDTA	Ethylenediaminetetraacetic acid
FIEB Foundation	Foundation for Research in Ethology and Biodiversity
GGT	Gamma–glutamyl transferase
GLDH	Glutamate dehydrogenase
IFCC-CLSI guidelines	International Federation of Clinical Chemistry and Laboratory Medicine and the Clinical and Laboratory Standards Institute guidelines
MCH	Mean corpuscular hemoglobin
MCHC	Mean corpuscular hemoglobin concentration
MCV	Mean corpuscular volume
RBC	Red blood cell
RI	Reference interval
SARS-CoV-2	Severe Acute Respiratory Syndrome Coronavirus type 2
SEM	Standard error of the mean
WBC	White blood cell
