# Supplementary material for: Clinical Chemistry and Hematology Values in Captive European Mink (Mustela lutreola): Reference Intervals and Evaluation of the Effects of Sex, Age, and Body Condition
Source: Vet Sci. 2026 Jun 26;13(7):619. doi: 10.3390/vetsci13070619 (PMC13417968; doi:10.3390/vetsci13070619)
Supplement: Supplementary file 1 [file vetsci-13-00619-s001.zip › vetsci-4367856-supplementary.pdf]

**Table S1.** Hematological analytes of a single captive collection of European mink (*Mustela lutreola*) in Spain. For all analytes, the mean, standard error of the mean (SEM), median, minimum, maximum, and the 10th and 90th percentiles, as well as the lower and upper reference intervals, including their 90% confidence intervals (CI) between parentheses, were calculated for all animals. Outliers detected and retained in the analysis are also indicated (†) (**A: All animals**).

| **A (All animals)** | | | | | | | | | | | |
|---|---|---|---|---|---|---|---|---|---|---|---|
| **Analyte (Unit)** | **N** | **Mean** | **SEM** | **Median** | **Minimum** | **Maximum** | **10th Percentile** | **90th Percentile** | **Outliers retained†** | **Lower Reference Interval Limit (90% CI)** | **Upper Reference Interval Limit (90% CI)** |
| RBC (x10¹²/L) | 97 | 7.89 | 0.08 | 8.05 | 4.24 | 9.19 | 6.78 | 8.71 | 0 | 5.9 (4.2 – 6.5) | 9 (8.8 – 9.2) |
| Hemoglobin (g/L) | 97 | 155.27 | 1.69 | 159.00 | 80.00 | 181.00 | 139.60 | 169.20 | 0 | 92.2 (80 – 130.5) | 177.7 (172.6 – 181.0) |
| Hematocrit (%) | 97 | 51.20 | 0.61 | 52.00 | 28.00 | 63.00 | 44.00 | 58.00 | 0 | 33.9 (28 – 41.5) | 60.6 (58.6 – 63) |
| MCV (fL) | 54 | 66.63 | 0.96 | 66.40 | 49.80 | 79.60 | 58.50 | 77.30 | 0 | 50.1 (49.8 – 53.3) | 79.4 (78.3 – 79.6) |
| MCH (pg) | 54 | 19.74 | 0.24 | 19.85 | 9.90 | 22.60 | 18.70 | 21.30 | 0 | 12.2 (9.9 – 17.8) | 22.4 (21.4 - 22.6) |
| MCHC (g/L) | 54 | 301.04 | 3.38 | 298.50 | 267.00 | 379.00 | 275.50 | 325.50 | 4 | 267 (267 – 271.4) | 377.9 (356.6 – 379.0) |
| Platelets (x10⁹/L) | 84 | 513.12 | 15.41 | 520.00 | 65.00 | 883.00 | 341.00 | 690.50 | 0 | 103.1 (65.0 – 271.9) | 773.1 (740.0 – 883.0) |
| WBC (x10⁹/L) | 97 | 4.97 | 0.24 | 4.70 | 1.20 | 15.80 | 2.40 | 7.96 | 0 | 1.6 (1.2 – 1.9) | 11.2 (8.8 – 15.8) |
| Neutrophils (x10⁹/L) | 97 | 1.96 | 0.26 | 1.40 | 0.50 | 23.00 | 0.80 | 2.50 | 0 | 0.6 (0.5 – 0.7) | 10.2 ( 4.2 - 23.0) |
| Lymphocytes (x10⁹/L) | 97 | 2.78 | 0.15 | 2.40 | 0.60 | 7.80 | 1.10 | 4.80 | 0 | 0.6 (0.6 – 1.0) | 6.6 (5.3 – 7.8) |
| Monocytes (x10⁹/L) | 97 | 0.19 | 0.01 | 0.20 | 0.00 | .80 | 0.00 | 0.40 | 0 | 0.0 (0.0 – 0.0) | 0.6 (0.5 – 0.8) |
| Eosinophils (x10⁹/L) | 97 | 0.28 | 0.04 | 0.20 | 0.00 | 3.20 | 0.00 | 0.50 | 0 | 0.0 (0.0 – 0.0) | 1.1 (0.7 – 3.2) |
| Basophils (x10⁹/L) | 97 | 0.00 | 0.00 | 0.00 | 0.00 | 0.00 | 0.00 | 0.00 | 0 | 0.0 (0.0 – 0.0) | 0.0 (0.0 – 0.0) |
| Neutrophils (%) | 97 | 35.43 | 1.32 | 34.00 | 6.00 | 82.00 | 20.60 | 52.20 | 0 | 14.4 (6.0 – 17.0) | 73.3 (56.0 – 82) |
| Lymphocytes (%) | 97 | 55.13 | 1.36 | 57.00 | 13.00 | 81.00 | 36.00 | 71.20 | 0 | 13.5 (13.0 – 32.4) | 77.7 ( 73.6 – 81.0) |
| Monocytes (%) | 97 | 4.02 | 0.25 | 4.00 | 0.00 | 20.00 | 1.80 | 7.00 | 1 | 0.0 (0.0 – 1.0) | 8.6 (7.6 – 20.0) |
| Eosinophils (%) | 97 | 5.12 | 0.35 | 5.00 | 0.00 | 15.00 | 0.00 | 10.00 | 1 | 0.0 (0.0 – 0.0) | 13.7 (11.0 – 15.0) |
| Basophils (%) | 97 | 0.03 | 0.02 | 0.00 | 0.00 | 1.00 | 0.00 | 0.00 | 3 | 0.0 (0.0 – 0.0) | 1.0 (0.0 – 1.0) |

† *Outliers were retained following IFCC-CLSI guidelines and are indicated for the relevant parameters*

**Table S2**. Serum biochemical analytes of a single captive collection of European mink (*Mustela lutreola*) in Spain. For all analytes, the mean, standard error of the mean (SEM), median, minimum and maximum, and the 10th and 90th percentiles, were calculated for all animals. Lower and upper reference interval limits (including 90% confidence intervals, CI) are shown where applicable. For analytes with small sample size (N < 20), these interval estimates should be interpreted as preliminary due to limited statistical power (‡). Outliers detected and retained in the analysis are also indicated (†) (**A: All animals**).

| **A (All animals)** | | | | | | | | | | | |
|---|---|---|---|---|---|---|---|---|---|---|---|
| **Analyte (Unit)** | **N** | **Mean** | **SEM** | **Median** | **Minimum** | **Maximum** | **10th Percentile** | **90th Percentile** | **Outliers retained†** | **Lower Reference Interval Limit (90% CI)** | **Upper Reference Interval Limit (90% CI)** |
| Glucose (mmol/L) | 7 | 8.23 | 1.01 | 8.55 | 4.90 | 12.98 | 4.90 | | 0 | 3.4 ‡ | 17.5 ‡ |
| Triglycerides (mmol/L) | 8 | 0.62 | 0.06 | 0.58 | 0.34 | 0.88 | 0.34 | | 0 | 0.2 ‡ | 1.1 ‡ |
| Cholesterol (mmol/L) | 100 | 5.17 | 0.08 | 5.10 | 3.40 | 7.70 | 4.20 | 6.30 | 0 | 3.8 (3.4 – 4.0) | 7.1 (6.6 – 7.0) |
| Fructosamine (µmol/L) | 96 | 120.71 | 3.90 | 113.5 | 82.00 | 366.00 | 92.70 | 145.00 | 0 | 87 (82.0 – 89.0) | 268.6 (155.0 – 366.0) |
| Total protein (g/L) | 104 | 52.28 | 0.39 | 52.00 | 43.00 | 62.00 | 47.00 | 57.50 | 0 | 45.6 (43.0 – 46.6) | 60.4 (60.0 – 62.0) |
| Albumin (g/L) | 104 | 28.52 | 0.31 | 28.50 | 19.00 | 38.00 | 25.00 | 32.00 | 0 | 23.0 (19.0 – 24.0) | 36.8 (33.4 – 38.0) |
| Globulin (g/L) | 104 | 23.76 | 0.34 | 23.00 | 17.00 | 35.00 | 20.00 | 29.00 | 0 | 18.0 (17.0 – 18.6) | 31.0 (30.0 – 35.0) |
| A:G ratio | 104 | 1.23 | 0.02 | 1.21 | 0.54 | 1.83 | 0.93 | 1.58 | 0 | 0.8 (0.5 – 0.9) | 1.8 (1.7 – 1.8) |
| ALT (U/L) | 109 | 121.35 | 6.64 | 101.0 | 46.00 | 555.00 | 68.00 | 196.00 | 0 | 53.5 (46.0 – 63.0) | 313.5 (230.5 – 555.0) |
| AST (U/L) | 108 | 55.26 | 4.65 | 38.50 | 19.00 | 431.00 | 25.00 | 87.20 | 0 | 20.0 (19.0 – 23.5) | 164.9 (106.4 – 431.0) |
| ALP (U/L) | 103 | 44.50 | 2.47 | 37.00 | 8.00 | 149.00 | 20.00 | 78.40 | 0 | 9.6 (8.0 – 17.0) | 119.4 (90.3 – 149.0) |
| GGT (U/L) | 86 | 4.79 | 0.73 | 3.00 | 0.00 | 42.00 | 0.00 | 11.00 | 0 | 0.00 (0.0 – 0.0) | 26.7 (17.7 – 42.0) |
| GLDH (U/L) | 52 | 0.88 | 0.10 | 0.70 | 0.00 | 3.20 | 0.10 | 1.87 | 0 | 0.00 (0.0 – 0.1) | 3.2 (2.4 – 3.2) |
| Total bilirubin (µmol/L) | 97 | 0.71 | 0.06 | 0.60 | 0.00 | 3.50 | 0.10 | 1.44 | 0 | 0.00 (0.0 – 0.1) | 2.5 (1.8 – 3.5) |
| CK (U/L) | 108 | 149.94 | 11.85 | 117.0 | 0.00 | 683.00 | 53.90 | 329.50 | 3 | 7.2 (0.0 – 40.4) | 533.7 (411.9 – 683.0) |
| Amylase (U/L) | 103 | 61.93 | 2.16 | 60.00 | 0.00 | 223.00 | 47.40 | 79.00 | 4 | 6.4 (0.0 – 42.8) | 95.8 (85.0 – 223.0) |
| Lipase (U/L) | 102 | 24.04 | 0.89 | 23.00 | 0.00 | 86.00 | 16.30 | 32.40 | 3 | 7.5 (0.00 – 15.0) | 40.3 (37.0 – 86.0) |
| Urea (mmol/L) | 109 | 10.88 | 0.33 | 10.30 | 5.70 | 22.30 | 7.00 | 15.70 | 0 | 5.9 (5.7 – 6.4) | 20.1 (17.0 – 22.3) |
| Creatinine (µmol/L) | 109 | 35.47 | 0.94 | 34.00 | 16.00 | 69.00 | 25.00 | 47.00 | 0 | 16.0 (16.0-21.8) | 59.8 (52.8 – 69.0) |
| Sodium (mmol/L) | 102 | 152.82 | 0.35 | 152.5 | 144.00 | 165.00 | 149.00 | 157.00 | 0 | 146.0 (144.0 – 147.6) | 164.4 (157.9-165.0) |
| Potassium (mmol/L) | 102 | 4.31 | 0.04 | 4.30 | 3.70 | 6.50 | 3.80 | 4.80 | 0 | 3.7 (3.7 – 3.7) | 5.4 (5.1 – 6.5) |
| Chloride (mmol/L) | 8 | 119.38 | 1.74 | 118.5 | 113.00 | 130.00 | 113.00 | | 0 | 106.2 ‡ | 130.3 ‡ |
| Calcium (mmol/L) | 103 | 2.15 | 0.02 | 2.20 | 1.50 | 2.50 | 1.90 | 2.30 | 0 | 1.8 (1.5 – 1.9) | 2.4 (2.4 – 2.5) |
| Phosphate (mmol/L) | 103 | 1.32 | 0.04 | 1.30 | 0.60 | 2.20 | 0.80 | 1.80 | 0 | 0.6 (0.6 – 0.7) | 2.2 (2.0 – 2.2) |
| Magnesium (mmol/L) | 100 | 0.82 | 0.01 | 0.80 | 0.40 | 1.10 | 0.70 | 1.00 | 0 | 0.6 (0.4 – 0.7) | 1.0 (1.0 – 1.1) |
| Iron (mmol/L) | 100 | 31.07 | 0.97 | 30.75 | 3.70 | 52.50 | 21.10 | 43.19 | 0 | 4.6 (3.7 – 16.0) | 51.7 (44.9 – 52.5) |
| Vitamin E (µmol/L) | 34 | 40.48 | 3.25 | 34.80 | 10.44 | 92.34 | 24.82 | 77.49 | 1 | 14.9 (12.2 – 19.4) | 92.6 (64.1 – 130.8) |

† *Outliers were retained following IFCC-CLSI guidelines and are indicated for the relevant parameters*

‡ *Reference intervals should be interpreted with caution as preliminary estimates in these cases.*

**Table S3.** Sex-, age- and body condition–related differences in hematology. Data are mean ± standard error of the mean. Statistical significance was set at $p < 0.05$.

| | | SEX | | | AGE | | | BODY CONDITION | | | |
|---|---|---|---|---|---|---|---|---|---|---|---|
| **Analyte (Unit)** | **N** | **Male** | **Female** | ***p* value** | **Juvenile** | **Adult** | ***p* value** | **N** | **Normal** | **Overweight** | ***p* value** |
| RBC (x10[12]/L) | 97 | 7.95 ± 0.11 | 7.82 ± 0.11 | 0.243 | 7.90 ± 0.1 | 7.87 ± 0.11 | 0.439 | 85 | 7.90 ± 0.1 | 8.22 ± 0.06 | 0.099 |
| Hemoglobin (g/L) | 97 | 157.67 ± 2.3 | 152.61 ± 2.45 | **0.031*** | 155.47 ± 2.04 | 154.84 ± 3.06 | 0.716 | 85 | 152.92 ± 2.5 | 161.88 ± 1.43 | **0.018*** |
| Hematocrit (%) | 97 | 52.14 ± 0.79 | 50.15 ± 0.91 | 0.076 | 51.18 ± 0.72 | 51.23 ± 1.11 | 0.843 | 85 | 49.77 ± 0.88 | 53.61 ± 0.74 | **0.005*** |
| MCV (fL) | 54 | 66.65 ± 1.34 | 66.61 ± 1.36 | 0.806 | 66.25 ± 1.05 | 68.13 ± 2.35 | 0.246 | 45 | 64.43 ± 1.28 | 65.37 ± 1.25 | 0.300 |
| MCH (pg) | 54 | 19.89 ± 0.21 | 19.54 ± 0.49 | 0.720 | 19.61 ± 0.29 | 20.23 ± 0.28 | 0.328 | 45 | 19.45 ± 0.21 | 19.33 ± 0.51 | 0.368 |
| MCHC (g/L) | 54 | 300.74 ± 4.93 | 301.43 ± 4.46 | 0.540 | 301.4 ± 3.67 | 299.64 ± 8.69 | 0.505 | 45 | 303.46 ± 5.15 | 304.86 ± 5.55 | 0.982 |
| Platelets (x10[9]/L) | 84 | 484.77 ± 18.39 | 542.85 ± 24.3 | **0.014*** | 498.65 ± 20.1 | 549.29 ± 18.09 | 0.108 | 72 | 541.93 ± 19.96 | 494.11 ± 22.05 | 0.122 |
| WBC (x10[9]/L) | 97 | 4.31 ± 0.27 | 5.70 ± 0.37 | **0.002*** | 5.20 ± 0.27 | 4.47 ± 0.44 | **0.047*** | 85 | 5.28 ± 0.37 | 4.68 ± 0.31 | 0.362 |
| Neutrophils (x10[9]/L) | 97 | 2.00 ± 0.43 | 1.92 ± 0.29 | 0.848 | 1.72 ± 0.12 | 2.48 ± 0.78 | 0.551 | 85 | 1.87 ± 0.26 | 1.47 ± 0.10 | 0.772 |
| Lymphocytes (x10[9]/L) | 97 | 2.34 ± 0.17 | 3.26 ± 0.22 | **0.003*** | 3.02 ± 0.19 | 2.26 ± 0.16 | **0.038*** | 85 | 2.98 ± 0.22 | 2.76 ± 0.21 | 0.708 |
| Monocytes (x10[9]/L) | 97 | 0.19 ± 0.19 | 0.19 ± 0.02 | 0.724 | 0.18 ±0.01 | 0.21 ± 0.02 | 0.423 | 85 | 0.21 ± 0.02 | 0.19 ± 0.02 | 0.929 |
| Eosinophils (x10[9]/L) | 97 | 0.24 ± 0.03 | 0.32 ± 0.07 | 0.267 | 0.29 ± 0.05 | 0.24 ± 0.03 | 0.915 | 85 | 0.23 ± 0.03 | 0.26 ± 0.03 | 0.213 |
| Basophils (x10[9]/L) | 97 | 0.00 ± 0.00 | 0.00 ± 0.00 | 1 | 0.00 ± 0.00 | 0.00 ± 0.00 | 1 | 85 | 0.00 ± 0.00 | 0.00 ± 0.00 | 1 |
| Neutrophils (%) | 97 | 38.10 ± 1.61 | 32.48 ± 2.06 | **0.008*** | 34.77 ± 1.6 | 36.84 ± 2.31 | 0.618 | 85 | 35.35 ± 2.01 | 32.36 ± 1.39 | 0.504 |
| Lymphocytes (%) | 97 | 52.18 ±.1.49 | 58.41 ± 2.27 | **0.004*** | 56.23 ± 1.73 | 52.81 ± 2.13 | 0.147 | 85 | 56.10 ± 1.97 | 57.76 ± 1.49 | 0.914 |
| Monocytes (%) | 97 | 4.63 ± 0.41 | 3.35 ± 0.25 | **0.009*** | 3.76 ± 0.33 | 4.58 ± 0.33 | **0.011*** | 85 | 4.06 ± 0.42 | 4.36 ± 0.28 | 0.069 |
| Eosinophils (%) | 97 | 5.20 ± 0.51 | 5.03 ± 0.47 | 0.765 | 4.82 ± 0.4 | 5.74 ± 0.67 | 0.144 | 85 | 4.50 ± 0.48 | 5.67 ± 0.56 | 0.128 |
| Basophils (%) | 97 | 0.06 ± 0.03 | 0.00 ± 0.00 | 0.096 | 0.02 ± 0.01 | 0.06 ± 0.45 | 0.193 | 85 | 0.02 ± 0.02 | 0.06 ± 0.04 | 0.317 |

*Non-parametric Mann–Whitney unpaired tests were performed. P values in bold and with an asterisk (*) denote significant differences.*

**Table S4.** Hematological analytes for which significant differences between sex (males vs. females) and age class (juveniles vs. adults) were detected in a captive population of European mink (*Mustela lutreola*) in Spain (see Table S3). The specific mean, standard error of the mean (SEM), median, minimum, maximum, and the 10th and 90th percentiles, as well as the lower and upper reference intervals, were additionally calculated for each sex (**B: Males vs. Females**) and age (**C: Juveniles vs. Adults**) separately.

| **B (Males vs. Females)** | | | | | | | | | | | | |
|---|---|---|---|---|---|---|---|---|---|---|---|---|
| **Analyte (Unit)** | **Sex** | **N** | **Mean** | **SEM** | **Median** | **Minimum** | **Maximum** | **10th Percentile** | **90th Percentile** | **Outliers retained†** | **Lower Reference Interval Limit** | **Upper Reference Interval Limit** |
| Hemoglobin (g/L) | Male | 51 | 157.67 | 2.30 | 162.00 | 89.00 | 181.00 | 145.20 | 171.00 | 2 | 91.1 | 180.4 |
| | Female | 46 | 152.61 | 2.45 | 156.50 | 80.00 | 176.00 | 137.70 | 167.30 | 0 | 84.2 | 175.3 |
| Platelets (x10$^9$/L) | Male | 43 | 484.77 | 18.39 | 499.00 | 97.00 | 693.00 | 370.40 | 632.80 | 0 | 101.9 | 692.5 |
| | Female | 41 | 542.85 | 24.36 | 558.00 | 65.00 | 883.00 | 304.00 | 753.80 | 2 | 74.1 | 877.5 |
| WBC (x10$^9$/L) | Male | 51 | 4.31 | 0.27 | 4.00 | 1.20 | 10.90 | 2.00 | 6.44 | 0 | 1.3 | 10.3 |
| | Female | 46 | 5.70 | 0.38 | 5.50 | 2.00 | 15.80 | 2.68 | 8.39 | 0 | 2.0 | 15.0 |
| Lymphocytes (x10$^9$/L) | Male | 51 | 2.34 | 0.17 | 2.20 | 0.60 | 6.80 | 1.02 | 3.86 | 0 | 0.6 | 6.4 |
| | Female | 46 | 3.26 | 0.23 | 3.25 | 1.10 | 7.80 | 1.17 | 5.09 | 0 | 1.1 | 7.5 |
| Neutrophils (%) | Male | 51 | 38.10 | 1.61 | 36.00 | 13.00 | 80.00 | 25.00 | 53.00 | 0 | 16.0 | 73.4 |
| | Female | 46 | 32.48 | 2.06 | 30.00 | 6.00 | 82.00 | 17.00 | 47.20 | 2 | 7.8 | 79.0 |
| Lymphocytes (%) | Male | 51 | 52.18 | 1.49 | 56.00 | 14.00 | 68.00 | 36.00 | 63.00 | 0 | 20.0 | 68.0 |
| | Female | 46 | 58.41 | 2.28 | 59.00 | 13.00 | 81.00 | 40.80 | 74.30 | 0 | 13.0 | 80.7 |
| Monocytes (%) | Male | 51 | 4.63 | 0.41 | 4.00 | 1.00 | 20.00 | 2.20 | 7.80 | 0 | 1.0 | 16.7 |
| | Female | 46 | 3.35 | 0.26 | 3.00 | 0.00 | 8.00 | 1.00 | 6.00 | 0 | 0.0 | 7.8 |
| **C (Juveniles vs. Adults)** | | | | | | | | | | | | |
| **Analyte (Unit)** | **Age** | **N** | **Mean** | **SEM** | **Median** | **Minimum** | **Maximum** | **10% Percentile** | **90% Percentile** | **Outliers retained†** | **Lower Reference Interval Limit** | **Upper Reference Interval Limit** |
| WBC (x10$^9$/L) | Juvenile | 66 | 5.20 | 0.27 | 5.10 | 1.20 | 11.40 | 2.48 | 8.36 | 0 | 1.5 | 11.1 |
| | Adult | 31 | 4.47 | 0.44 | 4.10 | 1.60 | 15.80 | 2.40 | 6.16 | 0 | 1.8 | 11.8 |
| Lymphocytes (x10$^9$/L) | Juvenile | 66 | 3.02 | 0.19 | 2.55 | 0.60 | 7.80 | 1.10 | 5.09 | 0 | 0.6 | 7.1 |
| | Adult | 31 | 2.26 | 0.16 | 2.20 | 0.70 | 4.00 | 1.02 | 3.58 | 0 | 0.6 | 4.5 |
| Monocytes (%) | Juvenile | 66 | 3.76 | 0.33 | 3.00 | 0.00 | 20.00 | 1.00 | 6.30 | 5 | 0.0 | 12.6 |
| | Adult | 31 | 4.58 | 0.33 | 4.00 | 1.00 | 8.00 | 2.00 | 7.80 | 0 | 1.2 | 8.90 |

† *Outliers were retained following IFCC-CLSI guidelines and are indicated for the relevant parameters*

**Table S5.** Sex-, age- and body condition–related differences in serum biochemistry. Data are mean ± standard error of the mean. Statistical significance was set at $p < 0.05$.

| | | SEX | | | AGE | | | | BODY CONDITION | | |
|---|---|---|---|---|---|---|---|---|---|---|---|
| **Analyte (Unit)** | **N** | **Male** | **Female** | ***p* value** | **Juvenile** | **Adult** | ***p* value** | **N** | **Normal** | **Overweight** | ***p* value** |
| Glucose (mmol/L) | 7 | 7.11 ± 0.81 | 11.05 ± 1.93 | 0.121 | 8.49 ± 1.16 | 6.69 | 0.617 | 7 | 9.00 ± 2.33 | 7.66 ± 0.77 | 0.724 |
| Triglycerides (mmol/L) | 8 | 0.60 ± 0.08 | 0.65 ± 0.12 | 0.880 | 0.58 ± 0.07 | 0.73 ± 0.15 | 0.238 | 8 | 0.65 ± 0.08 | 0.59 ± 0.10 | 0.770 |
| Cholesterol (mmol/L) | 100 | 4.78 ± 0.09 | 5.61 ± 0.12 | **< 0.001*** | 5.14 ± 0.96 | 5.23 ± 0.17 | 0.994 | 90 | 5.29 ± 0.12 | 4.88 ± 0.12 | **0.034*** |
| Fructosamine (µmol/L) | 96 | 128.87 ± 6.46 | 110.21 ± 2.50 | **0.011*** | 122.15 ± 5.45 | 117.68 ± 3.93 | 0.944 | 86 | 117.96 ± 5.95 | 119.82 ± 3.06 | 0.105 |
| Total protein (g/L) | 104 | 51.65 ± 0.46 | 52.98 ± 0.65 | 0.099 | 51.93 ± 0.50 | 53.00 ± 0.62 | 0.104 | 94 | 52.56 ± 0.62 | 51.65 ± 0.53 | 0.359 |
| Albumin (g/L) | 104 | 28.63 ± 0.36 | 28.06 ± 0.52 | 0.078 | 29.09 ± 0.38 | 27.35 ± 0.50 | **0.020*** | 94 | 28.61 ± 0.48 | 28.75 ± 0.43 | 0.606 |
| Globulin (g/L) | 104 | 22.73 ± 0.39 | 24.96 ± 0.53 | **0.005*** | 22.84 ± 0.36 | 25.65 ± 0.62 | **< 0.001*** | 94 | 23.94 ± 0.51 | 22.90 ± 0.46 | 0.287 |
| A:G ratio | 104 | 1.30 ± 0.03 | 1.15 ± 0.03 | **< 0.001*** | 1.30 ± 0.03 | 1.09 ± 0.04 | **< 0.001*** | 94 | 1.23 ± 0.03 | 1.29 ± 0.04 | 0.212 |
| ALT (U/L) | 109 | 141.08 ± 10.57 | 98.06 ± 5.97 | **< 0.001*** | 118.16 ± 6.80 | 128.09 ± 15.0 | 0.894 | 98 | 119.98 ± 10.14 | 123.15 ± 10.40 | 0.376 |
| AST (U/L) | 108 | 61.53 ± 7.90 | 47.71 ± 3.66 | 0.127 | 61.92 ± 6.58 | 41.37 ± 3.30 | **0.023*** | 98 | 50.12 ± 3.79 | 51.88 ± 5.93 | 0.745 |
| ALP (U/L) | 103 | 38.05 ± 3.02 | 51.90 ± 3.78 | **0.002*** | 47.90 ± 3.13 | 37.62 ± 3.77 | **0.016*** | 93 | 45.84 ± 3.50 | 42.35 ± 4.12 | 0.405 |
| GGT (U/L) | 86 | 5.25 ± 0.98 | 4.23 ± 1.11 | 0.606 | 4.93 ± 0.90 | 4.50 ± 1.28 | 0.263 | 76 | 3.44 ± 0.72 | 4.19 ± 0.94 | 0.920 |
| GLDH (U/L) | 52 | 0.89 ± 0.11 | 0.87 ± 0.19 | 0.336 | 0.79 ± 0.11 | 1.05 ± 0.19 | 0.294 | 44 | 0.92 ± 0.17 | 0.87 ± 0.15 | 0.485 |
| Total bilirubin (µmol/L) | 97 | 0.79 ± 0.09 | 0.61 ± 0.08 | 0.097 | 0.70 ± 0.08 | 0.75 ± 0.08 | 0.187 | 87 | 0.58 ± 0.07 | 0.68 ± 0.09 | 0.330 |
| CK (U/L) | 108 | 151.51 ± 17.34 | 148.04 ± 15.87 | 0.904 | 157.96 ± 14.34 | 133.20 ± 21.03 | 0.051 | 98 | 149.71 ± 16.19 | 160.03 ± 20.37 | 0.342 |
| Amylase (U/L) | 103 | 61.14 ± 3.75 | 62.87 ± 1.61 | 0.119 | 62.20 ± 3.08 | 61.38 ± 1.96 | 0.830 | 93 | 57.41 ± 2.21 | 66.81 ± 4.61 | 0.239 |
| Lipase (U/L) | 102 | 24.36 ± 1.60 | 23.66 ± 0.53 | 0.655 | 23.38 ± 1.22 | 25.35 ± 1.09 | 0.143 | 92 | 22.80 ± 0.90 | 25.54 ± 2.01 | 0.563 |
| Urea (mmol/L) | 109 | 9.77 ± 0.38 | 12.18 ± 0.50 | **< 0.001*** | 10.44 ± 0.38 | 11.79 ± 0.62 | 0.070 | 98 | 10.71 ± 0.48 | 10.76 ± 0.49 | 0.767 |
| Creatinine (µmol/L) | 109 | 40.51 ± 1.18 | 29.52 ± 1.00 | **< 0.001*** | 34.26 ± 1.22 | 38.03 ± 1.34 | **0.014*** | 98 | 34.00 ± 1.33 | 35.68 ± 1.25 | 0.225 |
| Sodium (mmol/L) | 102 | 153.65 ± 0.45 | 151.85 ± 0.52 | **0.016*** | 152.85 ± 0.37 | 152.76 ± 0.76 | 0.532 | 93 | 152.25 ± 0.48 | 153.70 ± 0.50 | **0.045*** |
| Potassium (mmol/L) | 102 | 4.46 ± 0.06 | 4.12 ± 0.05 | **< 0.001*** | 4.28 ± 0.06 | 4.35 ± 0.07 | 0.282 | 93 | 4.29 ± 0.06 | 4.32 ± 0.05 | 0.512 |
| Chloride (mmol/L) | 8 | 119.40 ± 2.84 | 119.33 ± 1.2 | 0.549 | 118.00 ± 1.15 | 123.50 ± 6.5 | 0.615 | 0 | | | |
| Calcium (mmol/L) | 103 | 2.16 ± 0.02 | 2.14 ± 0.03 | 0.875 | 2.18 ± 0.02 | 2.10 ± 0.03 | **0.015*** | 92 | 2.14 ± 0.02 | 2.21 ± 0.02 | 0.084 |
| Phosphate (mmol/L) | 103 | 1.38 ± 0.06 | 1.23 ± 0.05 | **0.047*** | 1.31 ± 0.05 | 1.33 ± 0.07 | 0.938 | 92 | 1.23 ± 0.05 | 1.39 ± 0.06 | 0.069 |
| Magnesium (mmol/L) | 100 | 0.83 ± 0.01 | 0.80 ± 0.02 | 0.302 | 0.83 ± 0.01 | 0.81 ± 0.02 | 0.413 | 90 | 0.80 ± 0.02 | 0.85 ± 0.02 | **0.044*** |
| Iron (mmol/L) | 100 | 32.37 ± 1.36 | 29.55 ± 1.37 | 0.123 | 31.41 ± 1.14 | 30.38 ± 1.86 | 0.692 | 90 | 29.56 ± 1.31 | 32.34 ± 1.60 | 0.168 |
| Vitamin E (µmol/L) | 34 | 35.55 ± 4.07 | 44.85 ± 4.84 | 0.058 | 35.92 ± 3.00 | 48.84 ± 6.98 | **0.044*** | 34 | 43.09 ± 4.23 | 36.26 ± 5.05 | 0.235 |

*Non-parametric Mann–Whitney unpaired tests were performed. P values in bold and with an asterisk (*) denote significant differences.*

**Table S6.** Serum biochemical analytes for which significant differences between sex (males vs. females) were detected in a captive population of European mink (*Mustela lutreola*) in Spain (see Table S5). The specific mean, SEM, median, minimum, maximum, and the 10th and 90th percentiles, as well as the lower and upper reference intervals, were additionally calculated for each sex (**B: Males *vs.* Females**) separately. Outliers detected and retained in the analysis are also indicated (†).

| | | | | | | | | | | | | |
|---|---|---|---|---|---|---|---|---|---|---|---|---|
| **B (Males vs. Females)** | | | | | | | | | | | | |
| **Analyte (Unit)** | **Sex** | **N** | **Mean** | **SEM** | **Median** | **Minimum** | **Maximum** | **10th Percentile** | **90th Percentile** | **Outliers retained†** | **Lower Reference Interval Limit** | **Upper Reference Interval Limit** |
| Cholesterol (mmol/L) | Male | 53 | 4.78 | 0.09 | 4.70 | 3.40 | 7.20 | 4.00 | 5.50 | 0 | 3.5 | 6.8 |
| | Female | 47 | 5.61 | 0.12 | 5.70 | 4.00 | 7.70 | 4.58 | 6.62 | 0 | 4.0 | 7.6 |
| Fructosamine (μmol/L) | Male | 54 | 128.87 | 6.46 | 117.00 | 87.00 | 366.00 | 99.50 | 156.50 | 0 | 88.1 | 351.0 |
| | Female | 42 | 110.21 | 2.50 | 110.50 | 82.00 | 151.00 | 89.00 | 132.70 | 0 | 82.4 | 150.0 |
| Globulin (g/L) | Male | 55 | 22.73 | 0.39 | 23.00 | 17.00 | 30.00 | 19.00 | 26.40 | 0 | 17.4 | 29.6 |
| | Female | 49 | 24.96 | 0.53 | 24.00 | 18.00 | 35.00 | 21.00 | 30.00 | 0 | 18.3 | 34 |
| A:G ratio | Male | 55 | 1.30 | 0.03 | 1.34 | 0.76 | 1.76 | 1.01 | 1.61 | 0 | 0.8 | 1.7 |
| | Female | 49 | 1.15 | 0.03 | 1.12 | 0.54 | 1.83 | 0.89 | 1.45 | 0 | 0.6 | 1.8 |
| ALT (U/L) | Male | 59 | 141.08 | 10.57 | 111.00 | 70.00 | 555.00 | 78.00 | 210.00 | 0 | 73 | 494.5 |
| | Female | 50 | 98.06 | 5.97 | 86.00 | 46.00 | 245.00 | 62.10 | 153.60 | 6 | 47.7 | 239.8 |
| ALP (U/L) | Male | 55 | 38.05 | 3.02 | 34.00 | 8.00 | 135.00 | 17.00 | 62.00 | 0 | 8.4 | 124.6 |
| | Female | 48 | 51.90 | 3.78 | 43.00 | 11.00 | 149.00 | 25.80 | 83.60 | 0 | 12.6 | 138.2 |
| Urea (mmol/L) | Male | 59 | 9.77 | 0.38 | 9.30 | 6.00 | 22.30 | 6.80 | 14.50 | 0 | 6 | 18.8 |
| | Female | 50 | 12.18 | 0.50 | 11.75 | 5.70 | 21.30 | 7.37 | 16.98 | 0 | 5.7 | 20.9 |
| Creatinine (μmol/L) | Male | 59 | 40.51 | 1.18 | 40.00 | 26.00 | 69.00 | 30.00 | 52.00 | 0 | 26.0 | 68.5 |
| | Female | 50 | 29.52 | 1.00 | 29.00 | 16.00 | 55.00 | 21.10 | 38.80 | 4 | 16.0 | 50.9 |
| Sodium (mmol/L) | Male | 55 | 153.65 | 0.46 | 153.00 | 149.00 | 165.00 | 150.00 | 157.00 | 0 | 149.0 | 165.0 |
| | Female | 47 | 151.85 | 0.52 | 152.00 | 144.00 | 164.00 | 147.00 | 155.20 | 0 | 144.4 | 163.0 |
| Potassium (mmol/L) | Male | 55 | 4.46 | 0.06 | 4.40 | 3.70 | 6.50 | 3.96 | 5.08 | 0 | 3.7 | 6.0 |
| | Female | 47 | 4.12 | 0.05 | 4.10 | 3.70 | 5.60 | 3.70 | 4.52 | 0 | 3.7 | 5.5 |
| Phosphate (mmol/L) | Male | 56 | 1.38 | 0.06 | 1.40 | 0.60 | 2.20 | 0.77 | 1.86 | 0 | 0.6 | 2.2 |
| | Female | 47 | 1.23 | 0.05 | 1.20 | 0.70 | 2.20 | 0.78 | 1.62 | 0 | 0.7 | 2.1 |

† *Outliers were retained following IFCC-CLSI guidelines and are indicated for the relevant parameters*

**Table S7.** Serum biochemical analytes for which significant differences between age class (juveniles vs. adults) were detected in a captive population of European mink (*Mustela lutreola*) in Spain (see Table S5). The specific mean, SEM, median, minimum, maximum, and the 10th and 90th percentiles, were calculated for each category of age (**C: Juveniles *vs.* Adults**) separately. Lower and upper reference interval limits are shown where applicable. For analytes with small sample size (N < 20), these interval estimates should be interpreted as preliminary due to limited statistical power (‡). Outliers detected and retained in the analysis are also indicated (†).

| C (Juveniles vs. Adults) | | | | | | | | | | | | |
|---|---|---|---|---|---|---|---|---|---|---|---|---|
| **Analyte (Unit)** | **Age** | **N** | **Mean** | **SEM** | **Median** | **Minimum** | **Maximum** | **10th Percentile** | **90th Percentile** | **Outliers retained†** | **Lower Reference Interval Limit** | **Upper Reference Interval Limit** |
| Albumin (g/L) | Juvenile | 70 | 29.09 | 0.38 | 29.00 | 23.00 | 38.00 | 25.00 | 33.00 | 0 | 23.8 | 38.0 |
| | Adult | 34 | 27.35 | 0.5 | 28.00 | 19.00 | 32.00 | 24.00 | 31.00 | 0 | 20.5 | 32.5 |
| Globulin (g/L) | Juvenile | 70 | 22.84 | 0.36 | 23.00 | 17.00 | 31.00 | 19.00 | 26.90 | 0 | 17.8 | 30.2 |
| | Adult | 34 | 25.65 | 0.62 | 25.00 | 19.00 | 35.00 | 21.00 | 30.00 | 0 | 19.0 | 33.9 |
| A:G ratio | Juvenile | 70 | 1.30 | 0.03 | 1.29 | 0.89 | 1.83 | 1.01 | 1.63 | 0 | 0.9 | 1.8 |
| | Adult | 34 | 1.09 | 0.04 | 1.09 | 0.54 | 1.46 | 0.84 | 1.43 | 0 | 0.6 | 1.5 |
| AST (U/L) | Juvenile | 73 | 61.92 | 6.58 | 52.00 | 20.00 | 431.00 | 25.40 | 103.00 | 2 | 20.0 | 276.3 |
| | Adult | 35 | 41.37 | 3.30 | 33.00 | 19.00 | 87.00 | 24.60 | 82.80 | 0 | 15.7 | 76.6 |
| ALP (U/L) | Juvenile | 69 | 47.90 | 3.13 | 42.00 | 8.00 | 149.00 | 24.00 | 82.00 | 0 | 8.8 | 138.5 |
| | Adult | 34 | 37.62 | 3.77 | 31.00 | 10.00 | 109.00 | 17.00 | 73.00 | 0 | 0.0 | 78.4 |
| Creatinine (µmol/L) | Juvenile | 74 | 34.26 | 1.22 | 33.00 | 16.00 | 69.00 | 23.00 | 45.50 | 0 | 16.0 | 68.1 |
| | Adult | 35 | 38.03 | 1.34 | 39.00 | 22.00 | 55.00 | 27.60 | 49.40 | 0 | 21.4 | 54.5 |
| Calcium (mmol/L) | Juvenile | 69 | 2.18 | 0.02 | 2.20 | 1.70 | 2.50 | 2.00 | 2.30 | 0 | 1.8 | 2.4 |
| | Adult | 34 | 2.10 | 0.03 | 2.10 | 1.50 | 2.40 | 1.90 | 2.30 | 0 | 1.6 | 2.4 |
| Vitamin E (µmol/L) | Juvenile | 22 | 35.92 | 3.00 | 32.71 | 20.65 | 90.48 | 24.55 | 48.56 | 0 | 20.9 | 73.0 |
| | Adult | 12 | 48.84 | 6.98 | 43.62 | 10.44 | 92.34 | 16.43 | 91.50 | 0 | 6.6 ‡ | 114.2 ‡ |

† *Outliers were retained following IFCC-CLSI guidelines and are indicated for the relevant parameters*

‡ *Reference intervals should be interpreted with caution as preliminary estimates in these cases.*
